# The 3-Month Effectiveness of a Stratified Blended Physiotherapy Intervention in Patients With Nonspecific Low Back Pain: Cluster Randomized Controlled Trial

**DOI:** 10.2196/31675

**Published:** 2022-02-25

**Authors:** Tjarco Koppenaal, Martijn F Pisters, Corelien JJ Kloek, Remco M Arensman, Raymond WJG Ostelo, Cindy Veenhof

**Affiliations:** 1 Research Group Empowering Healthy Behaviour Department of Health Innovations and Technology Fontys University of Applied Sciences Eindhoven Netherlands; 2 Center for Physical Therapy Research and Innovation in Primary Care Julius Health Care Centers Utrecht Netherlands; 3 Physical Therapy Research, Department of Rehabilitation, Physiotherapy Science and Sport University Medical Center Utrecht Brain Center Utrecht University Utrecht Netherlands; 4 Research Group Innovation of Human Movement Care Research Center Healthy and Sustainable Living HU University of Applied Sciences Utrecht Netherlands; 5 Department of Health Sciences Faculty of Science, VU University Amsterdam Amsterdam Movement Sciences research institute Amsterdam Amsterdam Netherlands; 6 Department of Epidemiology and Data Science Amsterdam University Medical Centre, Location VUmc Amsterdam Netherlands

**Keywords:** eHealth, nonspecific low back pain, physiotherapy, blended care, mobile phone

## Abstract

**Background:**

Patient education, home-based exercise therapy, and advice on returning to normal activities are established physiotherapeutic treatment options for patients with nonspecific low back pain (LBP). However, the effectiveness of physiotherapy interventions on health-related outcomes largely depends on patient self-management and adherence to exercise and physical activity recommendations. e-Exercise LBP is a recently developed stratified blended care intervention comprising a smartphone app integrated with face-to-face physiotherapy treatment. Following the promising effects of web-based applications on patients’ self-management skills and adherence to exercise and physical activity recommendations, it is hypothesized that e-Exercise LBP will improve patients’ physical functioning.

**Objective:**

This study aims to investigate the short-term (3 months) effectiveness of stratified blended physiotherapy (e-Exercise LBP) on physical functioning in comparison with face-to-face physiotherapy in patients with nonspecific LBP.

**Methods:**

The study design was a multicenter cluster randomized controlled trial with intention-to-treat analysis. Patients with nonspecific LBP aged ≥18 years were asked to participate in the study. The patients were treated with either stratified blended physiotherapy or face-to-face physiotherapy. Both interventions were conducted according to the Dutch physiotherapy guidelines for nonspecific LBP. Blended physiotherapy was stratified according to the patients’ risk of developing persistent LBP using the Keele STarT Back Screening Tool. The primary outcome was physical functioning (Oswestry Disability Index, range 0-100). Secondary outcomes included pain intensity, fear-avoidance beliefs, and self-reported adherence. Measurements were taken at baseline and at the 3-month follow-up.

**Results:**

Both the stratified blended physiotherapy group (104/208, 50%) and the face-to-face physiotherapy group (104/208, 50%) had improved clinically relevant and statistically significant physical functioning; however, there was no statistically significant or clinically relevant between-group difference (mean difference −1.96, 95% CI −4.47 to 0.55). For the secondary outcomes, stratified blended physiotherapy showed statistically significant between-group differences in fear-avoidance beliefs and self-reported adherence. In patients with a high risk of developing persistent LBP (13/208, 6.3%), stratified blended physiotherapy showed statistically significant between-group differences in physical functioning (mean difference −16.39, 95% CI −27.98 to −4.79) and several secondary outcomes.

**Conclusions:**

The stratified blended physiotherapy intervention e-Exercise LBP is not more effective than face-to-face physiotherapy in patients with nonspecific LBP in improving physical functioning in the short term. For both stratified blended physiotherapy and face-to-face physiotherapy, within-group improvements were clinically relevant. To be able to decide whether e-Exercise LBP should be implemented in daily physiotherapy practice, future research should focus on the long-term cost-effectiveness and determine which patients benefit most from stratified blended physiotherapy.

**Trial Registration:**

ISRCTN Registry 94074203; https://doi.org/10.1186/ISRCTN94074203

**International Registered Report Identifier (IRRID):**

RR2-https://doi.org/10.1186/s12891-020-3174-z

## Introduction

Low back pain (LBP)–related disability and the related socioeconomic burden remain high despite the many treatment options and health care resources available for LBP [1]. LBP can be caused by a specific pathology or trauma; however, in >90% of cases, an underlying disease is absent [2]. The clinical course of this so-called *nonspecific LBP* varies and, as expected, is often less favorable; some patients recover within a couple of days or weeks, and other patients experience persistent disabling symptoms leading to chronic LBP. Up to 65% of primary care patients with LBP still experience pain 1 year after onset [3,4].

Clinical practice guidelines recommend a patient-centered approach for the management of LBP [5,6]. This approach identifies patients with an increased likelihood of delayed recovery at an early stage and stratifies the treatment accordingly [6-8]. An example of a tool for identifying individuals at risk of delayed recovery is the Keele STarT Back Screening Tool [9,10]. In general, in patients who have a *low risk* for delayed recovery, early management comprises advice, reassurance, and education about the nonspecific nature of their LBP and encouragement to stay active. For individuals at *medium risk* for developing persistent LBP, personalized and supervised exercise therapy should be considered. For the *high-risk* group, this exercise therapy can be supported by a graded activity approach or cognitive behavioral components [8,11]. In addition to a patient-centered and stratified approach, patients’ adherence to prescribed (home-based) exercises and recommended physical activity behavior is crucial for the effectiveness of care [12]. Earlier research showed that 45% to 70% of patients do not adhere to prescribed exercises and physical activity recommendations, whereas adherent patients with LBP have a reduced risk of recurrent LBP [13,14].

Within the treatment of patients with LBP, *blended care* is a promising new and understudied field [15]. Blended care refers to the integration of web-based and offline components within the treatment process and requires that both components contribute equally to the treatment process [16,17]. The integration of web-based components, such as websites and apps, provides new solutions to monitor and coach patients’ individual health behaviors and support the optimization of face-to-face care tailored to the patients’ individual needs [18-20]. Thereafter, web-based components can be an effective means of stimulating adherence to prescribed exercises at home between face-to-face sessions and possibly increase self-management of LBP [21,22]. Until now, evidence on patient-centered and stratified care has not been integrated into blended care. Therefore, we recently developed e-Exercise LBP, a stratified blended intervention in which a smartphone app is integrated within face-to-face physiotherapy treatment, and established its feasibility and proof of concept for the treatment of functional disability and pain [23]. e-Exercise LBP is an adapted version of previously developed and evaluated blended physiotherapy programs [24,25]. Following the promising effects of web-based applications for patients’ self-management skills and adherence to exercise and physical activity recommendations, it is hypothesized that e-Exercise LBP will improve patients’ physical functioning. However, the effectiveness of e-Exercise LBP in comparison with primary care physiotherapy still needs to be determined. The primary aim of this study is to investigate the short-term (3 months) effectiveness of stratified blended physiotherapy (e-Exercise LBP) on physical functioning in comparison with face-to-face physiotherapy in patients with nonspecific LBP.

## Methods

### Design and Ethical Considerations

The e-Exercise LBP study was a prospective multicenter cluster randomized controlled trial. The study protocol was approved by the medical research ethics committee of the University Medical Center Utrecht, the Netherlands (18-085/D), and registered at the onset of patient enrollment (ISRCTN 94074203). From January 2018 to June 2018, 122 physiotherapists working in 58 primary care physiotherapy practices were recruited and randomized to either stratified blended physiotherapy (e-Exercise LBP) or face-to-face physiotherapy. Details of the design and methods of the study have been published previously [26]. This study is reported according to the CONSORT (Consolidated Standards of Reporting Trials) statement for cluster randomized trials (Multimedia Appendix 1).

### Recruitment

#### Setting and Randomization

Physiotherapists were recruited by an invitational letter sent to the professional network of the authors and physiotherapists who participated in a previous e-Exercise study [24]. In addition, an advertisement was placed in the web-based newsletter of the Royal Dutch Society for Physiotherapy. Physiotherapy practices could participate with ≥1 physiotherapist, regardless of professional experience and education or specialization (eg, manual therapy). Physiotherapists were cluster randomized at the level of practice to avoid contamination. Treatment allocation was concealed and performed by an independent researcher using a computer-generated, a priori created, random sequence table and in a 1:1 allocation ratio. Physiotherapists and patients were not blinded to the group allocation.

The physiotherapists in the stratified blended physiotherapy group received two 4-hour training sessions on e-Exercise LBP and the study procedures. In the face-to-face physiotherapy group, physiotherapists received a 4-hour training session in current best practices according to the LBP guidelines of the Royal Dutch Society for Physiotherapy [11] and the study procedures.

#### Patients

Patients with LBP who contacted a participating physiotherapy practice were orally informed about the study and invited to participate. Interested patients received a patient information letter by email and an informative phone call by one of the researchers (TK or RMA) before the first appointment. When a patient was willing to participate after the phone call, a face-to-face appointment was scheduled (by TK or RMA) to obtain written informed consent and verify eligibility. The eligibility criteria were as follows: (1) being a patient requesting physiotherapy treatment for nonspecific LBP, defined as pain in the lumbosacral region (sometimes associated with radiating pain to the buttock or leg) [11]; (2) aged ≥18 years; (3) possessing a smartphone or tablet (iOS or Android operating system) with access to the internet; and (4) mastery of the Dutch language. The exclusion criteria were as follows: (1) a specific cause of LBP determined through medical imaging or a medical physician, (2) serious comorbidities (eg, malignancy or stroke), and (3) current pregnancy because of the prevalence of pelvic girdle pain as a specific form of LBP.

### Intervention

#### Experimental: Stratified Blended Physiotherapy (e-Exercise LBP)

Patients allocated to the stratified blended physiotherapy group received blended physiotherapy, comprising a smartphone app integrated within face-to-face physiotherapy treatment [23,26]. Both the contents of the smartphone app and the face-to-face physiotherapy treatment are based on the recommendations of the LBP guidelines of the Royal Dutch Society for Physiotherapy [11]. The duration and content of the stratified blended physiotherapy intervention were based on the patients’ risk for developing persistent LBP (*low*, *medium,* or *high*) using the Keele STarT Back Screening Tool [9,10]. The smartphone app contains video-supported self-management information, video-supported exercises, and a goal-oriented physical activity module. Both the contents of face-to-face care and the smartphone app were tailored by the physiotherapists to the patients’ individual needs and progress (Table 1). Although physiotherapists were recommended to treat according to the stratified blended physiotherapy protocol, they were free to deviate from the protocol with respect to their clinical competence. Print screens of the smartphone app are provided in Multimedia Appendix 2.

**Table 1:** Overview of the stratified blended physiotherapy intervention (e-Exercise low back pain [LBP]).

Mode of delivery			Low-risk profile		Medium-risk profile		High-risk profile
**Smartphone app**							
	Duration	3 weeks		12 weeks		12 weeks	
	Information module	Knowledge-based platform with several LBP self-management information themes (directly available)		12 weekly self-management themes, including assignments		12 weekly self-management themes, including assignments, pain education, and psychosocial risk factors	
	Exercise module	3 to 4 home-based exercises tailored to the patient’s specific functional limitations		3 to 4 home-based exercises tailored to the patient’s specific functional limitations		3 to 4 home-based exercises tailored to the patient’s specific functional limitations	
	Physical activity module	Physical activity recommendations in accordance with the LBP guidelines of the Royal Dutch Association for Physiotherapy		A 3-day baseline test to determine the current level of physical activity; an 11-week, 3-times per week, goal-oriented training program to maintain or improve the level of physical activity; in patients avoiding physical activity because of LBP, a graded activity functionality can be activated		A 3-day baseline test to determine the current level of physical activity; an 11-week, 3-times per week, goal-oriented training program to maintain or improve the level of physical activity using a graded activity approach	
**Face-to-face care**							
	Sessions	2 sessions		Maximum of 8 sessions		Maximum of 12 sessions	
	Content	Reassurance, information about LBP, instruction on self-management options, and the importance of adequate physical activity behavior		Content similar to low risk, and in addition, the physiotherapist can consider providing evidence-based interventions (eg, passive or active joint mobilization) as recommended by guideline LBP of the Royal Dutch Association for Physiotherapy		Content similar to medium risk, and in addition, the physiotherapist will address the patient’s specific psychosocial risk factors using a cognitive behavioral approach, and pain education will be given	
**Integration of face-to-face care and smartphone app**							
	First session	Provide information about LBP and instructions on home-based exercises addressing patient’s specific functional limitations using the smartphone app		Provide information about LBP, instructions on home-based exercises addressing patient’s specific functional limitations, and instructions on 3-day baseline test using the smartphone app		Provide information about LBP, instructions on home-based exercises addressing patient’s specific functional limitations, and instructions on 3-day baseline test using the smartphone app	
	Middle sessions	N/A^a^		Evaluation of progress with the smartphone app and optimizing face-to-face care		Evaluation of progress with the smartphone app and optimizing face-to-face care	
	Final session	Evaluate the progress with the smartphone app and give recommendations to prevent recurrent episodes of LBP and maintain or improve the physical activity level		Evaluate the progress with the smartphone app and give recommendations to prevent recurrent episodes of LBP and maintain or improve the physical activity level		Evaluate the progress with the smartphone app and give recommendations to prevent recurrent episodes of LBP and maintain or improve the physical activity level	

^a^N/A: not applicable.

#### Control: Face-to-face Physiotherapy

Patients in the face-to-face physiotherapy group received only face-to-face care following the recommendations of the LBP guidelines of the Royal Dutch Society for Physiotherapy [11]. The guideline distinguishes between three different patient profiles based on the clinical course of recovery (ie, normal recovery, abnormal recovery without predominant psychosocial factors, and abnormal recovery with predominant psychosocial factors) but does not use a specific tool to stratify care a priori. The content of face-to-face physiotherapy was the same as the stratified blended care intervention (ie, information, exercises, and recommendations regarding physical activity). However, no recommendations or restrictions were provided with regard to the number of face-to-face sessions. Although web-based applications, such as websites and apps, are not recommended in the guidelines, physiotherapists were instructed to treat people without using any web-based applications to assure contrast between both groups. Practical content considerations were made by the physiotherapists themselves with respect to their clinical expertise.

### Measurements

Patients received a web-based questionnaire and an accelerometer at baseline and after 3 months of follow-up. Baseline measurements were conducted face to face and follow-up measurements through web-based communication (eg, FaceTime) or face to face when requested. No financial incentives were offered to complete the measurements. In the case of an unfilled questionnaire, patients were reminded after 7 and 14 days.

### Outcome Measures

#### Primary Outcome

*Physical functioning* because of pain was assessed using the Oswestry Disability Index (ODI; version 2.1a) [27,28]. The ODI was derived from the internationally accepted core outcome set for research into patients with nonspecific LBP [28]. A higher score (0-100) indicates increased functional disability.

#### Secondary Outcomes

*Pain intensity* was measured using an 11-point numeric rating scale for the average LBP intensity in the last week (0=no pain and 10=worst possible pain) [28,29].

*Physical activity* was objectively measured using Activ8 (2M Engineering) [30]. Patients were instructed to wear the Activ8 for 5 consecutive weeks starting at baseline and 8 consecutive days at the 3-month follow-up, except during sleeping, showering, bathing, or swimming. For the purpose of this study, only the first 7 days at both the baseline and 3-month follow-up were used. Accelerometer data were eligible if patients had worn the meter for at least 3 days for ≥10 hours a day [31]. For each patient, the mean time spent in moderate to vigorous physical activity (all activities >3.0 metabolic equivalents [32]) in minutes per day was computed by summation and divided by the number of eligible wearing days.

*Fear-avoidance beliefs about physical activity and work* were measured using the Fear-Avoidance Beliefs Questionnaire [33]. A higher score (range 0-96) indicates stronger fear and avoidance beliefs about how physical activity and work negatively affect LBP.

*Pain catastrophizing* was measured using the Pain Catastrophizing Scale [34]. A higher score (range 0-55) indicates a higher level of catastrophizing.

*Self-efficacy* was measured using the General Self-Efficacy Scale [35,36]. A higher score (range 10-40) indicates greater or stronger perceived self-efficacy.

*Self-management ability* was assessed using the Dutch version of the short form Patient Activation Measure [37]. A higher score (range 0-100) indicates a higher level of self-management.

*Health-related quality of life* was measured using the EuroQol-5D-5L [38]. A higher score (range 0-100) indicates a higher health-related quality of life.

*Patient self-reported adherence to prescribed home exercises* was measured using the Exercise Adherence Rating Scale [39]. A higher score (range 0-24) indicates better adherence.

#### Other Measures

Physiotherapists were asked to complete a registration form about the number of face-to-face sessions and report the applied treatment modalities per session. Patient characteristics and relevant clinical variables were assessed as part of the baseline questionnaire.

### Data Analysis

#### Overview

Descriptive statistics were used to explore baseline comparability and describe patients’ general characteristics, the number of face-to-face physiotherapy sessions, and the treatment modalities. To investigate selective attrition, general characteristics and primary baseline variables of dropouts and nondropouts were compared. All analyses were performed according to the *intention-to-treat* principle. Missing value analyses were performed by assuming the missing at random assumption. Multiple imputation was applied using multivariate imputation by chained equations with predictive mean matching for missing data in all outcomes. A total of 36 imputed data sets were generated, corresponding to the highest missing value percentage [40]. For all analyses, a 2-tailed significance level of *P*<.05 was considered statistically significant.

#### Analyses of Effectiveness

Linear mixed models (LMMs) with random effects to control for correlation within patients and physiotherapy practices [41] were used to determine the short-term effectiveness of stratified blended physiotherapy compared with face-to-face physiotherapy on primary and secondary outcome measures. Regression coefficients with 95% CIs signifying the differences between stratified blended physiotherapy and face-to-face physiotherapy were estimated. Analyses were adjusted for predefined confounders (eg, age, gender, and duration of pain [42-44]) that changed the between-group estimate by ≥10%. In addition, analyses were also adjusted for variables with a substantial difference at baseline that changed the regression coefficient for the between-group estimate by ≥10%. Potential interaction terms were explored. In the case of a statistically significant interaction term, stratified LMM analyses, controlling for the same variables as the primary analysis, were performed for the effect modifier.

#### Sample Size

The power calculation was based on the recommendations of Campbell et al [45] for cluster randomized trials and performed for the physical functioning primary outcome at the primary end point of the e-Exercise LBP study (ie, 12-month follow-up). In addition, repeated measures of the primary outcome during follow-up were taken into account [46]. An intraclass correlation coefficient of 0.05 was assumed. In addition, to detect a clinically relevant difference between groups at the 12-month follow-up, a difference of >6 points in physical functioning (ODI) [47,48], and an SD of 14.5 [49] were used in the sample size calculation. For the repeated measures of physical functioning, a correlation of 0.5 was estimated between baseline and follow-up measurements until the 12-month follow-up [46]. On the basis of these assumptions (power 80%; α=.05) and an average cluster size of 5, a total of 165 patients were needed. With an expected dropout rate of 20%, a total of 208 participating patients (n=104 per arm) were needed.

## Results

### Flow of Participants, Therapists, and Centers Through the Study

**Figure 1:**
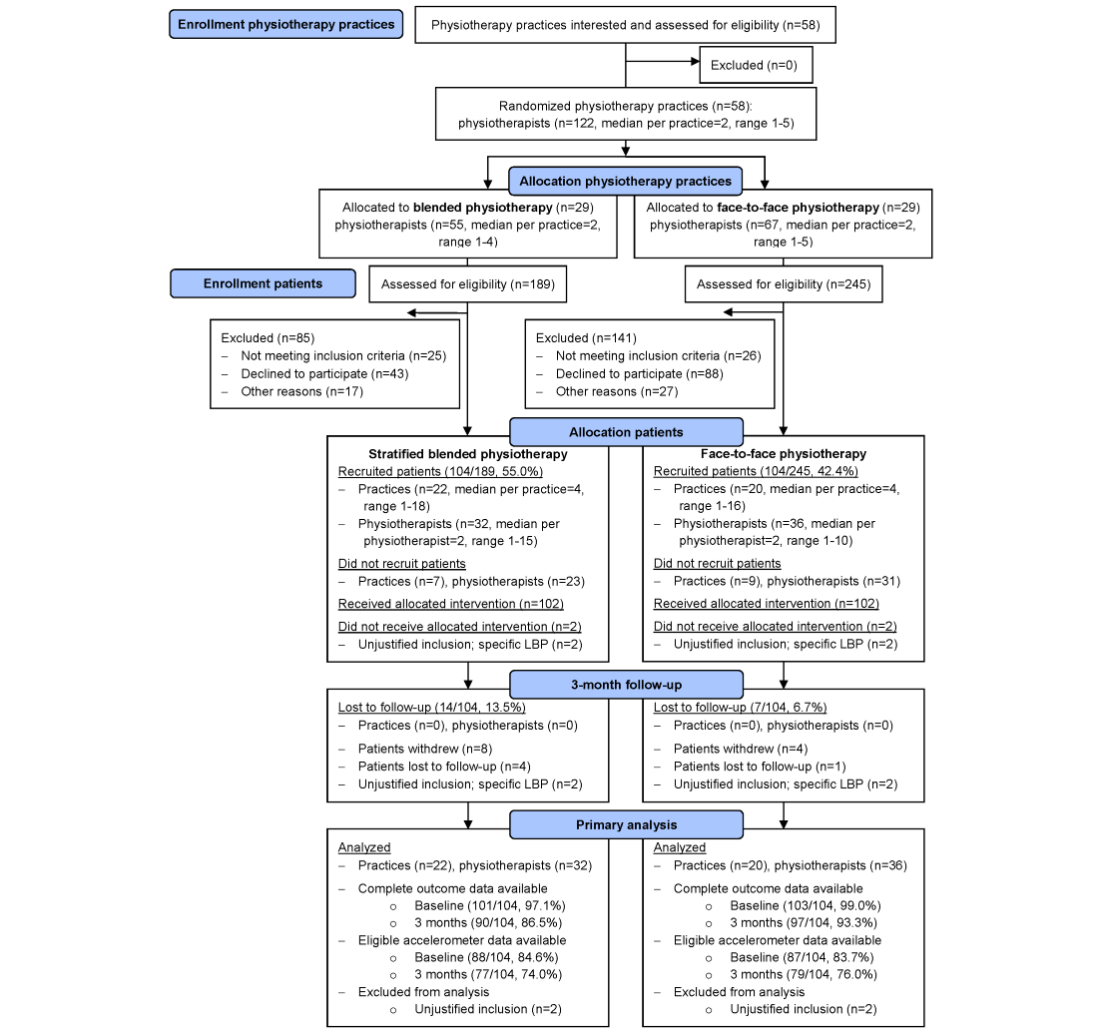
Flow diagram of the e-Exercise low back pain study.

From June 2018 to December 2019, 434 eligible patients with LBP were asked to participate in 58 physiotherapy practices. In 22 physiotherapy practices allocated to stratified blended physiotherapy and 20 practices allocated to face-to-face physiotherapy, 47.9% (208/434) patients were included (Figure 1).

Baseline characteristics of the patients are presented in Table 2. The stratified blended physiotherapy group comprised more men, more patients with a low level of education, and more patients with a duration of LBP >12 months. No other relevant differences in characteristics were seen between groups. At baseline, complete data on outcome measures were available from 97.1% (101/104) of the patients in the stratified blended physiotherapy group and 99% (103/104) of the patients in the face-to-face physiotherapy group, and eligible accelerometer data were available from 84.6% (88/104) and 83.7% (87/104), respectively. Of the 208 patients, 4 (1.9%) ineligible patients (n=2, 50% in the stratified blended physiotherapy group and 2, 50% in the face-to-face physiotherapy group) were unjustified included, did not receive the allocated intervention and were therefore excluded from all analyses.

At the 3-month follow-up, complete data on outcome measures were available from 86.5% (90/104) of the patients in the stratified blended physiotherapy group and 93.3% (97/104) of the patients in the face-to-face physiotherapy group, and eligible accelerometer data were available from 74% (77/104) and 76% (79/104) of these patients, respectively.

**Table 2:** Baseline demographic and clinical characteristics for patients from the stratified blended physiotherapy group and face-to-face physiotherapy group (N=208).

Characteristics			Baseline		
			Stratified blended physiotherapy (n=104)		Face-to-face physiotherapy (n=104)
Gender (female), n (%)			45 (43.3)		57 (54.8)
Age (years), mean (SD)			48.10 (15.08)		47.26 (13.58)
BMI (kg/m^2^), mean (SD)			25.78 (3.79)		26.31 (5.11)
Presence of comorbidities (yes), n (%)			38 (36.5)		28 (26.9)
**Past LBP^a^ surgery, n (%)**					
	None	100 (96.2)		101 (97.1)	
	Lumbar fusion	0 (0)		1 (1)	
	Lumbar discectomy	4 (3.9)		2 (1.9)	
Central sensitization (score 0-100), mean (SD)			30.88 (13.38)		30.17 (12.19)
**Educational level, n (%)**					
	Low	22 (21.2)		13 (12.5)	
	Middle	33 (31.7)		36 (34.6)	
	High	49 (47.1)		55 (52.9)	
**Duration of LBP complaints, n (%)**					
	0 to 6 weeks	37 (35.6)		49 (47.1)	
	6 to 12 weeks	11 (10.6)		19 (18.3)	
	12 weeks to 12 months	9 (8.7)		9 (8.7)	
	>12 months	47 (45.2)		27 (26)	
Physical functioning (score 0-100), mean (SD)			19.37 (15.64)		20.38 (13.99)
Pain intensity (average score 7 days 0-10), mean (SD)			5.61 (1.99)		5.36 (2.01)
Physical activity (MVPA^b^ minutes/day), mean (SD)			80.34 (36.75)		74.82 (40.94)
Health-related quality of life (score 0-100), mean (SD)			67.90 (18.08)		69.75 (17.63)
Fear-avoidance beliefs (score 0-96), mean (SD)			27.86 (16.03)		25.08 (16.18)
Pain catastrophizing (score 0-52), mean (SD)			11.06 (9.30)		10.21 (8.75)
Self-efficacy (score 10-40), mean (SD)			32.13 (4.36)		33.12 (3.62)
Patient activation (score 0-100), mean (SD)			62.48 (12.38)		64.75 (12.68)

^a^LBP: low back pain.

^b^MVPA: moderate to vigorous physical activity.

### Number and Treatment Modalities of Physiotherapy Sessions

In total, 189 physiotherapist registration forms were returned (n=95, 50.3% stratified blended physiotherapy and n=94, 49.7% in face-to-face physiotherapy). Table 3 shows the number and treatment modalities of the face-to-face physiotherapy sessions. Patients in the stratified blended physiotherapy group received an average of 4.81 (SD 2.94) face-to-face sessions. For the low-, medium-, and high-risk groups, the average number of sessions was 3.77 (SD 2.54), 5.65 (SD 2.65), and 7.67 (SD 3.54), respectively. Patients in the face-to-face physiotherapy group received an average of 4.94 (SD 2.26) face-to-face sessions. The average number of sessions for the *low-*, *medium-,* and *high*-*risk* groups was 4.88 (SD 2.02), 5.09 (SD 2.51), and 4.33 (SD 4.16), respectively.

In general, education was the main treatment modality during the face-to-face sessions in both treatment groups. No remarkable differences in treatment modalities were found between the 2 groups or between the different risk groups of developing persistent LBP.

**Table 3:** Number and treatment modalities of face-to-face physiotherapy sessions for patients from the stratified blended physiotherapy group and face-to-face physiotherapy group.

Category			Stratified blended physiotherapy (risk of developing persistent LBP^a^)									Face-to-face physiotherapy (risk of developing persistent LBP)						
			Low (n=52)		Medium (n=34)		High (n=9)		Total (n=95)		Low (n=57)			Medium (n=34)		High (n=3)		Total (n=94)
Number of sessions, mean (SD)			3.77 (2.54)		5.65 (2.65)		7.67 (3.54)		4.81 (2.94)		4.88 (2.02)			5.09 (2.51)		4.33 (4.16)		4.94 (2.26)
**Treatment modalities, n (%)^b^**																		
	Education	42 (81)		24 (71)		6 (67)		72 (76)		43 (75)			25 (74)		2 (67)		70 (74)	
	Strength exercises	9 (17)		3 (9)		1 (11)		13 (14)		7 (12)			6 (18)		0 (0)		13 (14)	
	Stability exercises	14 (27)		5 (15)		4 (44)		23 (24)		14 (25)			11 (32)		0 (0)		25 (27)	
	Endurance training	1 (2)		0 (0)		0 (0)		1 (1)		3 (5)			0 (0)		0 (0)		3 (3)	
	Functional exercises	3 (6)		0 (0)		0 (0)		3 (3)		4 (7)			0 (0)		0 (0)		4 (4)	
	Active mobilization	15 (29)		10 (29)		2 (22)		27 (28)		22 (39)			11 (32)		2 (67)		35 (37)	
	Passive mobilization	12 (23)		16 (47)		3 (33)		31 (33)		15 (26)			9 (26)		1 (33)		25 (27)	
	Massage	4 (8)		8 (24)		2 (22)		14 (15)		9 (19)			5 (15)		0 (0)		14 (15)	

^a^LBP: low back pain.

^b^Amount (%) of patients who received the treatment modality as part of the face-to-face physiotherapy session for ≥60% of the total number of face-to-face physiotherapy sessions.

### Is Stratified Blended Physiotherapy Effective Compared With Face-to-face Physiotherapy?

In the mixed model analyses, log likelihood ratios of naive models and models that included a random intercept for both physiotherapy practice and physiotherapist were similar. Therefore, physiotherapy practice or physiotherapist was not included as a level in the LMM analyses. At 3 months, LMM analyses showed no clinically relevant or statistically significant between-group difference in the primary outcome of physical functioning (mean difference [MD] −1.96, 95% CI −4.47 to 0.55). For the secondary outcomes, a statistically significant between-group difference was found in favor of stratified blended physiotherapy for fear-avoidance beliefs (MD −4.29, 95% CI −7.22 to −1.37) and patients’ self-reported adherence to prescribed home exercises (MD 0.73, 95% CI 0.06-1.39). Within-group analyses showed clinically relevant and statistically significant improvements in physical functioning (MD −11.48, 95% CI −15.06 to −7.91), average pain intensity (MD −2.38, 95% CI −3.00 to −1.76), and fear-avoidance beliefs (MD −5.14, 95% CI −9.22 to −1.06) in the stratified blended physiotherapy group. In the face-to-face physiotherapy group, clinically relevant and statistically significant improvements in physical functioning (MD −11.22, 95% CI −14.64 to −7.80) and average pain intensity (MD −2.51, 95% CI −3.11 to −1.90) were found (Table 4).

As indicated by a statistically significant interaction term, the patients’ risk of developing persistent LBP was an effect modifier of the between-group differences on the primary outcome of physical functioning. In patients with a high risk of developing persistent LBP, the stratified analysis showed a statistically significant between-group difference in favor of stratified blended physiotherapy on physical functioning (MD –16.39, 95% CI –27.98 to –4.79), average pain intensity (MD –3.43, 95% CI –6.55 to –0.31), and fear-avoidance beliefs (MD –14.51, 95% CI –28.21 to –0.81). In patients with a medium risk of developing persistent LBP, a statistically significant between-group difference was found in favor of stratified blended physiotherapy on fear-avoidance beliefs (MD –5.93, 95% CI –11.45 to –0.40). In patients with a low risk of developing persistent LBP, no statistically significant between-group differences were found (Table 5).

**Table 4:** Unadjusted and adjusted primary and secondary outcome measures: improvements and differences within and between groups (N=204).

Stratified blended physiotherapy (n=102)							Face-to-face physiotherapy (n=102)						Between group differences^a^				
Measurements, mean (SD)				Unadjusted within-group differences			Measurements, mean (SD)			Unadjusted within-group differences			Unadjusted			Adjusted^b^	
Baseline		3 months	Mean (95% CI)		*P* value	Baseline		3 months	Mean (95% CI)		*P* value	Mean (95% CI)		*P* value	Mean (95% CI)		*P* value
**Physical functioning (range 0-100)**																	
	19.39 (15.56)	7.91 (9.64)	−11.48 (−15.06 to −7.91)		<.001	20.20 (13.90)		8.97 (10.75)	−11.22 (−14.64 to −7.80)		<.001	−0.83 (−3.43 to 1.77)		.53	−1.98 (−4.49 to 0.53)		.12
**Pain intensity (average score 7 days; range 0-10)**																	
	5.67 (1.94)	3.29 (2.42)	−2.38 (−3.00 to −1.75)		<.001	5.40 (2.00)		2.90 (2.36)	−2.51 (−3.11 to −1.90)		<.001	0.31 (−0.35 to 0.98)		.36	0.08 (−0.57 to 0.74)		.80
**Physical activity (MVPA^c^ min/day)**																	
	81.97 (38.52)	78.58 (44.45)	−3.37 (−15.63 to 8.88)		.59	75.70 (41.89)		71.24 (40.34)	−4.42 (−16.91 to 8.07)		.49	3.49 (−8.38 to 15.36)		.56	3.62 (−8.27 to 15.51)		.55
**Fear-avoidance beliefs (range 0-96)**																	
	27.92 (16.01)	22.77 (13.38)	−5.14 (−9.25 to −1.04)		.01	25.51 (16.24)		24.82 (16.92)	−0.70 (−5.26 to 3.87)		.77	−3.73 (−6.63 to −0.82)		.01	−4.29 (−7.22 to −1.37)		<.001
**Pain catastrophizing (range 0-52)**																	
	11.02 (9.30)	8.97 (8.05)	−2.04 (−4.50 to 0.43)		.11	10.33 (8.76)		9.16 (9.84)	−1.17 (−3.74 to 1.40)		.37	−0.63 (−2.58 to 1.32)		.53	−0.96 (−2.95 to 1.02)		.34
**Self-efficacy (range 10-40)**																	
	32.05 (4.38)	32.02 (4.27)	−0.03 (−1.24 to 1.19)		.97	33.12 (3.63)		32.58 (3.99)	−0.54 (−1.59 to 0.52)		.32	0.12 (−0.82 to 1.06)		.81	0.14 (−0.82 to 1.10)		.77
**Health-related quality of life (range 0-100)**																	
	67.70 (18.09)	71.44 (20.07)	3.73 (−1.68 to 9.14)		.18	69.75 (17.71)		72.57 (21.06)	2.82 (−2.56 to 8.20)		.31	−0.65 (−6.38 to 5.08)		.82	0.95 (−4.80 to 6.69)		.75
**Patient activation (range 0-100)**																	
	62.43 (12.37)	62.45 (11.89)	0.02 (−3.42 to 3.46)		.99	64.72 (12.65)		64.39 (12.71)	−0.33 (−3.84 to 3.18)		.85	−0.83 (−3.94 to 2.27)		.60	−0.79 (−3.95 to 2.36)		.62
**Adherence to prescribed home exercises (range 0-24)^d^**																	
	N/A^e^	11.96 (2.43)	N/A		N/A	N/A		11.18 (2.17)	N/A		N/A	0.78 (0.13 to 1.44)		.02	0.73 (0.06 to 1.39)		.03

^a^Difference between baseline and 3 months in stratified blended physiotherapy versus face-to-face physiotherapy.

^b^Adjusted for baseline and duration of low back pain complaints (<12 vs >12 weeks).

^c^MVPA: moderate to vigorous physical activity.

^d^Patient self-reported adherence to prescribed home exercises could only be measured after the treatment period.

^e^N/A: not applicable.

**Table 5:** Adjusted primary and secondary outcome measures: improvements and differences between groups stratified for the risk of developing persistent low back pain (LBP; N=204).

Outcome measure	Risk of developing persistent LBP							
	Low risk (n=120)			Medium risk (n=71)			High risk (n=13)	
	Between-group difference, mean (95% CI)^a^	*P* value	Between-group difference, mean (95% CI)^a^		*P* value	Between-group difference, mean (95% CI)^a^		*P* value
Physical functioning (range 0-100)	−0.82 (−2.92 to 1.27)	.44	−3.48 (−8.99 to 2.03)		.22	−16.39 (−27.98 to −4.79)		.01
Pain intensity (average score 7 days; range 0-10)	0.30 (−0.52 to 1.13)	.47	0.01 (−1.08 to 1.11)		.98	−3.43 (−6.55 to −0.31)		.03
Physical activity (MVPA^b^ minutes/day)	3.80 (−12.05 to 19.65)	.64	1.08 (−16.70 to 18.86)		.91	39.50 (−1.24 to 80.24)		.06
Fear-avoidance beliefs (range 0-96)	−2.70 (−6.22 to 0.82)	.13	−5.93 (−11.45 to −0.40)		.04	−14.51 (−28.21 to −0.81)		.04
Pain catastrophizing (range 0-52)	0.28 (−2.03 to 2.59)	.81	−2.66 (−5.73 to 0.41)		.09	−14.47 (−31.89 to 2.94)		.10
Self-efficacy (range 10-40)	−0.58 (−1.76 to 0.60)	.33	0.85 (−0.92 to 2.62)		.35	1.50 (−4.02 to 7.02)		.60
Health-related quality of life (range 0-100)	1.26 (−7.15 to 9.68)	.77	0.84 (−6.47 to 8.15)		.82	15.84 (−3.92 to 35.61)		.12
Patient activation (range 0-100)	−2.22 (−6.38 to 1.93)	.29	1.85 (−3.27 to 6.97)		.48	7.49 (−1.35 to 16.34)		.10
Adherence to prescribed home exercises (range 0-24)	0.82 (−0.01 to 1.65)	.05	0.86 (−0.35 to 2.08)		.16	−1.19 (−3.37 to 0.99)		.28

^a^Difference between baseline and 3 months in stratified blended physiotherapy versus face-to-face physiotherapy per risk group and adjusted for baseline and duration of low back pain complaints (<12 vs >12 weeks).

^b^MVPA: moderate to vigorous physical activity.

## Discussion

### Principal Findings

This study evaluated the short-term (3 months) effectiveness of the stratified blended physiotherapy intervention e-Exercise LBP on physical functioning in comparison with face-to-face physiotherapy in patients with nonspecific LBP. In contrast to our expectations, the study results showed no statistically significant between-group difference in physical functioning and most of the secondary outcome measures. Only fear-avoidance beliefs and patient self-reported adherence to prescribed home exercises improved significantly in patients who were allocated to stratified blended physiotherapy. When looking at the different prognostic risk groups in patients with a high risk of developing persistent LBP, a statistically significant between-group difference in favor of stratified blended physiotherapy on physical functioning, average pain intensity, and fear-avoidance beliefs was found; however, these results come with some uncertainty.

### Interpretation of the Findings

The results of this study complement the findings from previous systematic reviews of randomized controlled trials that showed that in the short term, web-based applications could reduce LBP-related pain and disability; however, when compared with other interventions, the results are inconclusive [15,22,50]. A possible explanation for these inconclusive findings is the considerable heterogeneity in the studied characteristics and comparators, which hampers a clear comparison. For example, in our study, we integrated a web-based application within face-to-face guidance and compared it with face-to-face physiotherapy. Previous studies in this research area have focused predominantly on web-based applications as a stand-alone intervention without the face-to-face guidance of a health care professional [15,22,50]. Only a few studies have investigated web-based applications as an adjunct to face-to-face guidance, and the results regarding the added value of these combined interventions have been inconclusive [15,51]. Similar to our study, Sandal et al [51] investigated a smartphone app as an adjunct to face-to-face guidance. The app was tailored using artificial intelligence and did not influence face-to-face guidance. In this study, the reported between-group difference was statistically significant in favor of the combined intervention when compared with face-to-face guidance alone; however, the difference was small and of uncertain clinical significance.

Another example of heterogeneity in research on web-based applications is the large variation in delivery modes and duration. Similar to e-Exercise LBP, most web-based applications tailored the content of the intervention using patient characteristics and focused on self-management support, home-based exercise, and physical activity prescription [15,22,50]. However, the e-Exercise LBP app provided this content in weekly information modules and daily reminders to exercise and physical activity recommendations during a 3- or 12-week duration [26]; the duration in other studies ranged from 3 weeks to 1 year. In addition, the delivery modes showed large variation; that is, from no specific recommendations to multiple web- or telephone-based coaching sessions [15,22,50].

Thus, looking at the different characteristics of web-based applications, such as the role of the health care professional within the intervention and the delivery mode and duration, future research needs to focus on the comparison of web-based applications with different characteristics to obtain a better understanding of which elements work the best.

In our study, the short-term within-group improvements in physical functioning and average pain intensity of stratified blended physiotherapy were comparable with face-to-face physiotherapy, both of which were statistically significant and clinically meaningful. Patients in the stratified blended physiotherapy group improved on average 11.48 (95% CI −15.06 to −7.91) points (59.5%) in physical functioning, and patients in the face-to-face physiotherapy group improved by an average of 11.22 (95% CI −14.64 to −7.80) points (56%). For average pain intensity, these improvements were 2.38 (95% CI −3.00 to −1.76) points (42.8%) and 2.51 (95% CI −3.11 to −1.90) points (46.9%), respectively. As physical functioning and average pain intensity decreased by >30%, the improvements in both groups were considered clinically meaningful [52]. At the moment, e-Exercise LBP cannot be considered an alternative to face-to-face physiotherapy as this study was conducted as a superiority trial. To be able to value the true potential of e-Exercise LBP, the meaningful within-group improvements must be considered from the perspective of the additional effort and costs needed to implement such an intervention in daily physiotherapy practice. Future cost-effectiveness analyses will provide more insight into the long-term economic benefits of stratified blended physiotherapy. On the other hand, given the additional effort and costs, the potential of e-Exercise LBP needs to be considered from the perspective of future health care. It is expected that technology will be increasingly integrated into care for patients who are suitable to use it. Future studies need to determine which patients benefit most from a stratified blended physiotherapy approach.

The e-Exercise LBP intervention significantly increased patients’ self-reported adherence to prescribed home exercises, as hypothesized. In addition, it resulted in a significant reduction of fear-avoidance beliefs when compared with face-to-face physiotherapy. The between-group difference in patients’ self-reported adherence to prescribed home exercises was 3.3% points in favor of the e-Exercise LBP intervention. For fear-avoidance beliefs, the between-group difference was −4.6% points in favor of the e-Exercise LBP intervention. Although there are no established cutoffs for the minimum clinically important between-group differences in these outcomes, we consider the between-group differences as small. The difference in adherence might be explained by the benefits of integrating a smartphone app. The 24/7 availability of the app and functionality to remind the patient to perform scheduled exercises might have stimulated the patients to adhere to their prescribed home exercises in a better way than in the face-to-face physiotherapy group [18,53]. Further research on the long-term clinical relevance of adherence to home exercises as prescribed in e-Exercise LBP is ongoing.

The reduction of fear-avoidance beliefs complements evidence from a systematic review and meta-analysis that concluded that patient education provides reassurance for patients with acute or subacute LBP [54]. In our study, this reduction in the stratified blended physiotherapy group might be explained by the information module of the smartphone app. As the information module provides the patient with self-management information about LBP, the patient can reread the advice and reassurance given in the face-to-face sessions by the physiotherapist about their LBP at all times. As a result, the harmless and nonspecific nature of LBP is possibly remembered in a better way [55]. Long-term results should indicate whether this reduction in fear-avoidance beliefs also influences physical functioning, the handling of recurrent complaints, and costs a patient incurs because of LBP.

Several explanations are possible to clarify why the additional benefits of stratified blended physiotherapy were not found. A first explanation is that the added value of a stratified approach in itself must be critically evaluated. Although clinical practice guidelines have adopted and advocated a stratified care approach for several years to improve patient outcomes, the added value of this approach is, at present, unclear. On the basis of previous recommendations, we decided to use the Keele STarT Back Screening Tool to create a matched web-based application [10]. Our results show that, after specific training, treatment intensity (ie, the number of face-to-face sessions) in the e-Exercise LBP group was in line with the patient’s risk profile, which was not the case in our control group. However, this difference in treatment intensity did not lead to relevant between-group differences. This seems to be in line with more recent studies evaluating the stratified approach according to the Keele STarT Back Screening Tool. The results from these studies are not convincing regarding the added value of such a stratified approach [56,57]. Future research should focus on determining whether this concerns the added value of the tool itself or the added value of a stratified care approach in general.

In addition, stratified blended physiotherapy might not be suitable for every patient. Earlier research has shown that it is difficult to determine what works best for each individual patient [22,50]. In our study, we did not take into account the patient’s suitability for blended care to determine the optimal personalized blended treatment [58]. As a result, patients might have received stratified blended physiotherapy without being suitable for it; for example, a lack of motivation or digital literacy skills. Consequently, this could have resulted in the suboptimal effectiveness of our stratified blended physiotherapy intervention when compared with face-to-face physiotherapy. For future studies on blended care, it is recommended to use patients’ suitability for blended care as inclusion criteria or criteria to match treatment. The Dutch Blended Physiotherapy Checklist [58] could be a useful aid in this process.

A third explanation might be the relatively high proportion of patients with a low risk of developing persistent LBP in this study. For this group, earlier research has shown that providing advice as a single intervention is likely to reassure the patient with LBP but does not result in different management of pain and disability in the short term [54,59]. In addition, for this group, a stratified approach is beneficial from an economic perspective rather than in terms of clinical outcomes, as many of these patients recover completely within 2 to 3 weeks but nevertheless receive unnecessary treatment [57,60,61].

A final explanation is the timing of our follow-up measurement at 3 months only. Given the favorable course of LBP [62] and the rationale that stratified blended physiotherapy will stimulate patients’ self-management and adherence [21,22], patients in the stratified blended physiotherapy group might recover faster, which is not captured by a single follow-up measurement at 3 months. Therefore, for future studies that aim to investigate postintervention effectiveness, it is recommended to measure the clinical outcomes immediately after the intervention is completed and to monitor the time to recovery.

### Strengths and Limitations

This study had several important strengths. It is the next step in a multiphase development and implementation process based on the Center for eHealth Research Roadmap [63]. After developing a prototype and testing its feasibility in a pilot study [23], this study determined the short-term effectiveness of the final stratified blended physiotherapy protocol and showed its potential compared with face-to-face physiotherapy. The pragmatic, multicenter, cluster randomized controlled trial design allowed for the evaluation of stratified, blended physiotherapy in comparison with face-to-face physiotherapy in a real-world situation. The baseline characteristics of both treatment groups and the distribution of the different prognostic risk groups of developing persistent LBP reflect the characteristics of patients with LBP normally being treated in primary care physiotherapy [60], which enhances the generalizability of our results. The use of measurement instruments recommended in the core outcome set for research into patients with nonspecific LBP [28] and a low dropout rate (10.1%) guaranteed the internal validity of the results.

Nevertheless, this study also had a few limitations. First, the results seem to suggest that patients’ risk of developing persistent LBP could be an effect modifier of the between-group differences on the primary outcome. Especially in the highest risk group, consistent between-group differences were seen in both the primary and secondary outcomes, supporting the rationale for stratified blended physiotherapy. As it was not the primary aim of this study, the sample size calculation did not take interaction into account, the numbers were small, and therefore, the results should be interpreted with caution. Second, as we conducted a pragmatic study, the experiences of physiotherapists in either using web-based applications or treating patients with nonspecific LBP were not considered inclusion criteria for physiotherapy practices. However, given both the complexity of blended care [17] and the complexity of treating patients with nonspecific LBP [4], it can be expected that more experienced physiotherapists are able to deliver better treatment than less experienced physiotherapists. Therefore, experience might have influenced our analysis. Finally, 4 included patients were excluded from the analysis after being diagnosed with specific LBP. As this number is low and occurred equally in both treatment groups (2 in each group), we expect that this has not influenced the results [64].

### Conclusions

The stratified blended physiotherapy intervention e-Exercise LBP is not more effective than face-to-face physiotherapy in patients with nonspecific LBP in improving physical functioning in the short term. For both stratified blended physiotherapy and face-to-face physiotherapy, within-group improvements were clinically relevant. To be able to decide whether e-Exercise LBP should be implemented in daily physiotherapy practice, future research should focus on the long-term cost-effectiveness and determine which patients benefit most from stratified blended physiotherapy.

## Appendix

Multimedia Appendix 1 CONSORT-EHEALTH (Consolidated Standards of Reporting Trials of Electronic and Mobile Health Applications and Online Telehealth; version 1.6) checklist.

Multimedia Appendix 2 Print screens of the smartphone app.

## Abbreviations

CONSORT: Consolidated Standards of Reporting Trials
LBP: low back pain
LMM: linear mixed model
MD: mean difference
ODI: Oswestry Disability Index
