# Supplementary material for: The 3-Month Effectiveness of a Stratified Blended Physiotherapy Intervention in Patients With Nonspecific Low Back Pain: Cluster Randomized Controlled Trial
Source: J Med Internet Res. 2022 Feb 25;24(2):e31675. doi: 10.2196/31675 (PMC8917429; doi:10.2196/31675)
Supplement: Multimedia Appendix 1 [file jmir_v24i2e31675_app1.pdf]

CONSORT-EHEALTH (V 1.6.1) - Submission/Publication Form

The CONSORT-EHEALTH checklist is intended for authors of randomized trials evaluating web-based and Internet-based applications/interventions, including mobile interventions, electronic games (incl multiplayer games), social media, certain telehealth applications, and other interactive and/or networked electronic applications. Some of the items (e.g. all subitems under item 5 - description of the intervention) may also be applicable for other study designs.

The goal of the CONSORT EHEALTH checklist and guideline is to be
a) a guide for reporting for authors of RCTs,
b) to form a basis for appraisal of an ehealth trial (in terms of validity)

CONSORT-EHEALTH items/subitems are MANDATORY reporting items for studies published in the Journal of Medical Internet Research and other journals / scientific societies endorsing the checklist.

Items numbered 1., 2., 3., 4a., 4b etc are original CONSORT or CONSORT-NPT (non-pharmacologic treatment) items.
Items with Roman numerals (i., ii, iii, iv etc.) are CONSORT-EHEALTH extensions/clarifications.

As the CONSORT-EHEALTH checklist is still considered in a formative stage, we would ask that you also RATE ON A SCALE OF 1-5 how important/useful you feel each item is FOR THE PURPOSE OF THE CHECKLIST and reporting guideline (optional).

Mandatory reporting items are marked with a red *.
In the textboxes, either copy & paste the relevant sections from your manuscript into this form - please include any quotes from your manuscript in QUOTATION MARKS,
or answer directly by providing additional information not in the manuscript, or elaborating on why the item was not relevant for this study.

YOUR ANSWERS WILL BE PUBLISHED AS A SUPPLEMENTARY FILE TO YOUR PUBLICATION IN JMIR AND ARE CONSIDERED PART OF YOUR PUBLICATION (IF ACCEPTED).
Please fill in these questions diligently. Information will not be copyedited, so please use proper spelling and grammar, use correct capitalization, and avoid abbreviations.

DO NOT FORGET TO SAVE AS PDF _AND_ CLICK THE SUBMIT BUTTON SO YOUR ANSWERS ARE IN OUR DATABASE !!!

Citation Suggestion (if you append the pdf as Appendix we suggest to cite this paper in the caption):
Eysenbach G, CONSORT-EHEALTH Group
CONSORT-EHEALTH: Improving and Standardizing Evaluation Reports of Web-based and Mobile Health Interventions
J Med Internet Res 2011;13(4):e126
URL: http://www.jmir.org/2011/4/e126/
doi: 10.2196/jmir.1923
PMID: 22209829

Log in bij Google om je voortgang op te slaan. Meer informatie

*Vereist

Your name *

First Last

Tjarco Koppenaal

Primary Affiliation (short), City, Country *

University of Toronto, Toronto, Canada

Research Group Empowering Healthy Behaviou

Your e-mail address *

abc@gmail.com

t.koppenaal@fontys.nl

Title of your manuscript *

Provide the (draft) title of your manuscript.

The 3 Months Effectiveness of a Stratified Blended Physiotherapy Intervention in Patients with Nonspecific Low Back Pain: Cluster Randomized Controlled Trial

Name of your App/Software/Intervention *

If there is a short and a long/alternate name, write the short name first and add the long name in brackets.

e-Exercise Low Back Pain

Evaluated Version (if any)

e.g. "V1", "Release 2017-03-01", "Version 2.0.27913"

Jouw antwoord

Language(s) *

What language is the intervention/app in? If multiple languages are available, separate by comma (e.g. "English, French")

Dutch

URL of your Intervention Website or App

e.g. a direct link to the mobile app on app in appstore (itunes, Google Play), or URL of the website. If the intervention is a DVD or hardware, you can also link to an Amazon page.

https://www.e-exercise.nl/?page_id=172

URL of an image/screenshot (optional)

https://www.e-exercise.nl/?page_id=172

Accessibility *

Can an enduser access the intervention presently?

- ○ access is free and open
- ◉ access only for special usergroups, not open
- ○ access is open to everyone, but requires payment/subscription/in-app purchases
- ○ app/intervention no longer accessible
- ○ Anders:

Primary Medical Indication/Disease/Condition *

e.g. "Stress", "Diabetes", or define the target group in brackets after the condition, e.g. "Autism (Parents of children with)", "Alzheimers (Informal Caregivers of)"

Non-specific low back pain

Primary Outcomes measured in trial *

comma-separated list of primary outcomes reported in the trial

Physical functioning

Secondary/other outcomes

Are there any other outcomes the intervention is expected to affect?

Pain intensity, Physical activity, Fear avoidance beliefs about physical activity and work, Pain catastrophizing, Self-efficacy, Self-management ability, Health-related quality of life, Patient self-reported adherence to prescribed home exercises

Recommended "Dose" *

What do the instructions for users say on how often the app should be used?

- (x) Approximately Daily
- ( ) Approximately Weekly
- ( ) Approximately Monthly
- ( ) Approximately Yearly
- ( ) "as needed"
- ( ) Anders:

Approx. Percentage of Users (starters) still using the app as recommended after 3 months *

- (x) unknown / not evaluated
- ( ) 0-10%
- ( ) 11-20%
- ( ) 21-30%
- ( ) 31-40%
- ( ) 41-50%
- ( ) 51-60%
- ( ) 61-70%
- ( ) 71%-80%
- ( ) 81-90%
- ( ) 91-100%
- ( ) Anders:

Overall, was the app/intervention effective? *

- ( ) yes: all primary outcomes were significantly better in intervention group vs control
- ( ) partly: SOME primary outcomes were significantly better in intervention group vs control
- (x) no statistically significant difference between control and intervention
- ( ) potentially harmful: control was significantly better than intervention in one or more outcomes
- ( ) inconclusive: more research is needed
- ( ) Anders:

Article Preparation Status/Stage *

At which stage in your article preparation are you currently (at the time you fill in this form)

- ( ) not submitted yet - in early draft status
- ( ) not submitted yet - in late draft status, just before submission
- ( ) submitted to a journal but not reviewed yet
- ( ) submitted to a journal and after receiving initial reviewer comments
- (•) submitted to a journal and accepted, but not published yet
- ( ) published
- ( ) Anders:

Journal *

If you already know where you will submit this paper (or if it is already submitted), please provide the journal name (if it is not JMIR, provide the journal name under "other")

- ( ) not submitted yet / unclear where I will submit this
- (•) Journal of Medical Internet Research (JMIR)
- ( ) JMIR mHealth and UHealth
- ( ) JMIR Serious Games
- ( ) JMIR Mental Health
- ( ) JMIR Public Health
- ( ) JMIR Formative Research
- ( ) Other JMIR sister journal
- ( ) Anders:

Is this a full powered effectiveness trial or a pilot/feasibility trial? *

- ( ) Pilot/feasibility
- (•) Fully powered

Manuscript tracking number *

If this is a JMIR submission, please provide the manuscript tracking number under "other" (The ms tracking number can be found in the submission acknowledgement email, or when you login as author in JMIR. If the paper is already published in JMIR, then the ms tracking number is the four-digit number at the end of the DOI, to be found at the bottom of each published article in JMIR)

- ○ no ms number (yet) / not (yet) submitted to / published in JMIR
- ◉ Anders: #31675

TITLE AND ABSTRACT

1a) TITLE: Identification as a randomized trial in the title

1a) Does your paper address CONSORT item 1a? *

I.e does the title contain the phrase "Randomized Controlled Trial"? (if not, explain the reason under "other")

- ◉ yes
- ○ Anders:

1a-i) Identify the mode of delivery in the title

Identify the mode of delivery. Preferably use “web-based” and/or “mobile” and/or “electronic game” in the title. Avoid ambiguous terms like “online”, “virtual”, “interactive”. Use “Internet-based” only if Intervention includes non-web-based Internet components (e.g. email), use “computer-based” or “electronic” only if offline products are used. Use “virtual” only in the context of “virtual reality” (3-D worlds). Use “online” only in the context of “online support groups”. Complement or substitute product names with broader terms for the class of products (such as “mobile” or “smart phone” instead of “iphone”), especially if the application runs on different platforms.

	1	2	3	4	5	
subitem not at all important	○	◉	○	○	○	essential

Selectie wissen

Does your paper address subitem 1a-i? *

Copy and paste relevant sections from manuscript title (include quotes in quotation marks "like this" to indicate direct quotes from your manuscript), or elaborate on this item by providing additional information not in the ms, or briefly explain why the item is not applicable/relevant for your study

Stratified Blended Physiotherapy Intervention

1a-ii) Non-web-based components or important co-interventions in title

Mention non-web-based components or important co-interventions in title, if any (e.g., “with telephone support”).

	1	2	3	4	5	
subitem not at all important	◉	○	○	○	○	essential

Selectie wissen

Does your paper address subitem 1a-ii?

Copy and paste relevant sections from manuscript title (include quotes in quotation marks "like this" to indicate direct quotes from your manuscript), or elaborate on this item by providing additional information not in the ms, or briefly explain why the item is not applicable/relevant for your study

No, to keep title comprehensive

1a-iii) Primary condition or target group in the title

Mention primary condition or target group in the title, if any (e.g., “for children with Type I Diabetes”)
Example: A Web-based and Mobile Intervention with Telephone Support for Children with Type I Diabetes: Randomized Controlled Trial

	1	2	3	4	5	
subitem not at all important	○	○	○	○	◉	essential

Selectie wissen

Does your paper address subitem 1a-iii? *

Copy and paste relevant sections from manuscript title (include quotes in quotation marks "like this" to indicate direct quotes from your manuscript), or elaborate on this item by providing additional information not in the ms, or briefly explain why the item is not applicable/relevant for your study

Yes, "Patients with Non-specific Low Back Pain"

1b) ABSTRACT: Structured summary of trial design, methods, results, and conclusions

NPT extension: Description of experimental treatment, comparator, care providers, centers, and blinding status.

1b-i) Key features/functionalities/components of the intervention and comparator in the METHODS section of the ABSTRACT

Mention key features/functionalities/components of the intervention and comparator in the abstract. If possible, also mention theories and principles used for designing the site. Keep in mind the needs of systematic reviewers and indexers by including important synonyms. (Note: Only report in the abstract what the main paper is reporting. If this information is missing from the main body of text, consider adding it)

	1	2	3	4	5	
subitem not at all important	○	○	○	○	◉	essential

Selectie wissen

Does your paper address subitem 1b-i? *

Copy and paste relevant sections from the manuscript abstract (include quotes in quotation marks "like this" to indicate direct quotes from your manuscript), or elaborate on this item by providing additional information not in the ms, or briefly explain why the item is not applicable/relevant for your study

Yes, "The patients were treated with either stratified blended physiotherapy or face-to-face physiotherapy. Both interventions were conducted according to the Dutch physiotherapy guidelines for nonspecific LBP. Blended physiotherapy was stratified according to the patients' risk of developing persistent LBP using the Keele STarT Back Screening Tool."

1b-ii) Level of human involvement in the METHODS section of the ABSTRACT

Clarify the level of human involvement in the abstract, e.g., use phrases like “fully automated” vs. “therapist/nurse/care provider/physician-assisted” (mention number and expertise of providers involved, if any). (Note: Only report in the abstract what the main paper is reporting. If this information is missing from the main body of text, consider adding it)

	1	2	3	4	5	
subitem not at all important	○	○	●	○	○	essential

Selectie wissen

Does your paper address subitem 1b-ii?

Copy and paste relevant sections from the manuscript abstract (include quotes in quotation marks "like this" to indicate direct quotes from your manuscript), or elaborate on this item by providing additional information not in the ms, or briefly explain why the item is not applicable/relevant for your study

Partly, "E-Exercise LBP is a recently developed stratified blended care intervention consisting of a smartphone application integrated with face-to-face physiotherapy treatment."

1b-iii) Open vs. closed, web-based (self-assessment) vs. face-to-face assessments in the METHODS section of the ABSTRACT

Mention how participants were recruited (online vs. offline), e.g., from an open access website or from a clinic or a closed online user group (closed usergroup trial), and clarify if this was a purely web-based trial, or there were face-to-face components (as part of the intervention or for assessment). Clearly say if outcomes were self-assessed through questionnaires (as common in web-based trials). Note: In traditional offline trials, an open trial (open-label trial) is a type of clinical trial in which both the researchers and participants know which treatment is being administered. To avoid confusion, use “blinded” or “unblinded” to indicated the level of blinding instead of “open”, as “open” in web-based trials usually refers to “open access” (i.e. participants can self-enrol). (Note: Only report in the abstract what the main paper is reporting. If this information is missing from the main body of text, consider adding it)

	1	2	3	4	5	
subitem not at all important	●	○	○	○	○	essential

Selectie wissen

Does your paper address subitem 1b-iii?

Copy and paste relevant sections from the manuscript abstract (include quotes in quotation marks "like this" to indicate direct quotes from your manuscript), or elaborate on this item by providing additional information not in the ms, or briefly explain why the item is not applicable/relevant for your study

Partly, "Patients with nonspecific LBP aged 18 years and older were asked to participate in the study."

1b-iv) RESULTS section in abstract must contain use data

Report number of participants enrolled/assessed in each group, the use/uptake of the intervention (e.g., attrition/adherence metrics, use over time, number of logins etc.), in addition to primary/secondary outcomes. (Note: Only report in the abstract what the main paper is reporting. If this information is missing from the main body of text, consider adding it)

	1	2	3	4	5	
subitem not at all important	○	○	●	○	○	essential

Selectie wissen

Does your paper address subitem 1b-iv?

Copy and paste relevant sections from the manuscript abstract (include quotes in quotation marks "like this" to indicate direct quotes from your manuscript), or elaborate on this item by providing additional information not in the ms, or briefly explain why the item is not applicable/relevant for your study

Yes, "Both the stratified blended physiotherapy group (n=104) and the face-to-face physiotherapy group (n=104)"

1b-v) CONCLUSIONS/DISCUSSION in abstract for negative trials

Conclusions/Discussions in abstract for negative trials: Discuss the primary outcome - if the trial is negative (primary outcome not changed), and the intervention was not used, discuss whether negative results are attributable to lack of uptake and discuss reasons. (Note: Only report in the abstract what the main paper is reporting. If this information is missing from the main body of text, consider adding it)

	1	2	3	4	5	
subitem not at all important	○	○	○	●	○	essential

Selectie wissen

Does your paper address subitem 1b-v?

Copy and paste relevant sections from the manuscript abstract (include quotes in quotation marks "like this" to indicate direct quotes from your manuscript), or elaborate on this item by providing additional information not in the ms, or briefly explain why the item is not applicable/relevant for your study

Yes, "The stratified blended physiotherapy intervention e-Exercise LBP is not more effective than face-to-face physiotherapy in patients with nonspecific LBP to improve physical functioning in the short term."

INTRODUCTION

2a) In INTRODUCTION: Scientific background and explanation of rationale

2a-i) Problem and the type of system/solution

Describe the problem and the type of system/solution that is object of the study: intended as stand-alone intervention vs. incorporated in broader health care program? Intended for a particular patient population? Goals of the intervention, e.g., being more cost-effective to other interventions, replace or complement other solutions? (Note: Details about the intervention are provided in "Methods" under 5)

	1	2	3	4	5	
subitem not at all important	○	○	○	◉	○	essential

Selectie wissen

Does your paper address subitem 2a-i? *

Copy and paste relevant sections from the manuscript (include quotes in quotation marks "like this" to indicate direct quotes from your manuscript), or elaborate on this item by providing additional information not in the ms, or briefly explain why the item is not applicable/relevant for your study

Yes, "Low back pain-related disability and the related socioeconomic burden remain high despite the many treatment options and health care resources available for low back pain (LBP)[1]." and "Clinical practice guidelines recommend a patient-centred approach for the management of LBP[5,6]. This approach identifies patients with an increased likelihood of delayed recovery at an early stage and stratifies treatment accordingly[6–8]." and "n addition to a patient-centred and stratified approach, patients' adherence to prescribed (home-based) exercises and recommended physical activity behaviour is crucial for the effectiveness of care[12]." and "Within the treatment of patients with LBP, 'blended care' is a promising new and understudied field[15]."

2a-ii) Scientific background, rationale: What is known about the (type of) system

Scientific background, rationale: What is known about the (type of) system that is the object of the study (be sure to discuss the use of similar systems for other conditions/diagnoses, if appropiate), motivation for the study, i.e. what are the reasons for and what is the context for this specific study, from which stakeholder viewpoint is the study performed, potential impact of findings [2]. Briefly justify the choice of the comparator.

	1	2	3	4	5	
subitem not at all important	○	○	○	◉	○	essential

Selectie wissen

Does your paper address subitem 2a-ii? *

Copy and paste relevant sections from the manuscript (include quotes in quotation marks "like this" to indicate direct quotes from your manuscript), or elaborate on this item by providing additional information not in the ms, or briefly explain why the item is not applicable/relevant for your study

Yes, "Therefore, we recently developed e-Exercise LBP, a stratified blended intervention in which a smartphone application is integrated within face-to-face physiotherapy treatment, and established its feasibility and proof of concept for treatment of functional disability and pain[23]. E-Exercise LBP is an adapted version of previously developed and evaluated blended physiotherapy programs[24,25]. Following the promising effects of online applications for patients' self-management skills and adherence to exercise and physical activity recommendations, it is hypothesized that e-Exercise LBP will improve patients' physical functioning."

2b) In INTRODUCTION: Specific objectives or hypotheses

Does your paper address CONSORT subitem 2b? *

Copy and paste relevant sections from the manuscript (include quotes in quotation marks "like this" to indicate direct quotes from your manuscript), or elaborate on this item by providing additional information not in the ms, or briefly explain why the item is not applicable/relevant for your study

Yes, "it is hypothesized that e-Exercise LBP will improve patients' physical functioning." and "he primary aim of this study was to investigate the short-term (3 months) effectiveness of stratified blended physiotherapy (e-Exercise LBP) on physical functioning in comparison to face-to-face physiotherapy in patients with nonspecific LBP."

METHODS

3a) Description of trial design (such as parallel, factorial) including allocation ratio

Does your paper address CONSORT subitem 3a? *

Copy and paste relevant sections from the manuscript (include quotes in quotation marks "like this" to indicate direct quotes from your manuscript), or elaborate on this item by providing additional information not in the ms, or briefly explain why the item is not applicable/relevant for your study

Yes, "The e-Exercise LBP study was a prospective, multicentre cluster randomized controlled trial."

3b) Important changes to methods after trial commencement (such as eligibility criteria), with reasons

Does your paper address CONSORT subitem 3b? *

Copy and paste relevant sections from the manuscript (include quotes in quotation marks "like this" to indicate direct quotes from your manuscript), or elaborate on this item by providing additional information not in the ms, or briefly explain why the item is not applicable/relevant for your study

Not applicable, no important changes made.

3b-i) Bug fixes, Downtimes, Content Changes

Bug fixes, Downtimes, Content Changes: ehealth systems are often dynamic systems. A description of changes to methods therefore also includes important changes made on the intervention or comparator during the trial (e.g., major bug fixes or changes in the functionality or content) (5-iii) and other "unexpected events" that may have influenced study design such as staff changes, system failures/downtimes, etc. [2].

	1	2	3	4	5	
subitem not at all important	○	◉	○	○	○	essential

Selectie wissen

Does your paper address subitem 3b-i?

Copy and paste relevant sections from the manuscript (include quotes in quotation marks "like this" to indicate direct quotes from your manuscript), or elaborate on this item by providing additional information not in the ms, or briefly explain why the item is not applicable/relevant for your study

Not applicable, described in the development study.

4a) Eligibility criteria for participants

Does your paper address CONSORT subitem 4a? *

Copy and paste relevant sections from the manuscript (include quotes in quotation marks "like this" to indicate direct quotes from your manuscript), or elaborate on this item by providing additional information not in the ms, or briefly explain why the item is not applicable/relevant for your study

Yes, "Eligibility criteria were as follows: (i) being a patient requesting physiotherapy treatment for nonspecific LBP, defined as pain in the lumbosacral region (sometimes associated with radiating pain to the buttock or leg)[11], (ii) age 18 years or older, (iii) possessing a smartphone or tablet (iOS or Android operating system) with access to the internet, and (iv) mastery of the Dutch language. Exclusion criteria were as follows: (i) a specific cause of LBP determined through medical imaging or a medical doctor, (ii) serious comorbidities (e.g., malignancy, stroke), and (iii) current pregnancy because of the prevalence of pelvic girdle pain as a specific form of LBP."

4a-i) Computer / Internet literacy

Computer / Internet literacy is often an implicit “de facto” eligibility criterion - this should be explicitly clarified.

	1	2	3	4	5	
subitem not at all important	(●)	○	○	○	○	essential

Selectie wissen

Does your paper address subitem 4a-i?

Copy and paste relevant sections from the manuscript (include quotes in quotation marks "like this" to indicate direct quotes from your manuscript), or elaborate on this item by providing additional information not in the ms, or briefly explain why the item is not applicable/relevant for your study

No, "possessing a smartphone or tablet (iOS or Android operating system) with access to the internet"

4a-ii) Open vs. closed, web-based vs. face-to-face assessments:

Open vs. closed, web-based vs. face-to-face assessments: Mention how participants were recruited (online vs. offline), e.g., from an open access website or from a clinic, and clarify if this was a purely web-based trial, or there were face-to-face components (as part of the intervention or for assessment), i.e., to what degree got the study team to know the participant. In online-only trials, clarify if participants were quasi-anonymous and whether having multiple identities was possible or whether technical or logistical measures (e.g., cookies, email confirmation, phone calls) were used to detect/prevent these.

	1	2	3	4	5	
subitem not at all important	○	○	○	●	○	essential

Selectie wissen

Does your paper address subitem 4a-ii? *

Copy and paste relevant sections from the manuscript (include quotes in quotation marks "like this" to indicate direct quotes from your manuscript), or elaborate on this item by providing additional information not in the ms, or briefly explain why the item is not applicable/relevant for your study

Yes, "Patients with LBP who contacted a participating physiotherapy practice were orally informed about the study and invited to participate. Interested patients received a patient information letter by e-mail and an informative phone call by one of the researchers (TK or RA) prior to the first appointment. When a patient was willing to participate after the phone call, a face-to-face appointment was scheduled (by TK or RA) to obtain written informed consent and verify eligibility." and "Patients received an online questionnaire and an accelerometer at baseline and after 3 months of follow-up. Baseline measurement was conducted face to face and follow-up measurement through online communication, e.g., FaceTime, or face to face when requested."

4a-iii) Information giving during recruitment

Information given during recruitment. Specify how participants were briefed for recruitment and in the informed consent procedures (e.g., publish the informed consent documentation as appendix, see also item X26), as this information may have an effect on user self-selection, user expectation and may also bias results.

	1	2	3	4	5	
subitem not at all important	○	○	●	○	○	essential

Selectie wissen

Does your paper address subitem 4a-iii?

Copy and paste relevant sections from the manuscript (include quotes in quotation marks "like this" to indicate direct quotes from your manuscript), or elaborate on this item by providing additional information not in the ms, or briefly explain why the item is not applicable/relevant for your study

Yes, "Patients with LBP who contacted a participating physiotherapy practice were orally informed about the study and invited to participate. Interested patients received a patient information letter by e-mail and an informative phone call by one of the researchers (TK or RA) prior to the first appointment. When a patient was willing to participate after the phone call, a face-to-face appointment was scheduled (by TK or RA) to obtain written informed consent and verify eligibility."

4b) Settings and locations where the data were collected

Does your paper address CONSORT subitem 4b? *

Copy and paste relevant sections from the manuscript (include quotes in quotation marks "like this" to indicate direct quotes from your manuscript), or elaborate on this item by providing additional information not in the ms, or briefly explain why the item is not applicable/relevant for your study

Yes, "Patients received an online questionnaire and an accelerometer at baseline and after 3 months of follow-up. Baseline measurement was conducted face to face and follow-up measurement through online communication, e.g., FaceTime, or face to face when requested."

4b-i) Report if outcomes were (self-)assessed through online questionnaires

Clearly report if outcomes were (self-)assessed through online questionnaires (as common in web-based trials) or otherwise.

	1	2	3	4	5	
subitem not at all important	○	○	●	○	○	essential

Selectie wissen

Does your paper address subitem 4b-i? *

Copy and paste relevant sections from the manuscript (include quotes in quotation marks "like this" to indicate direct quotes from your manuscript), or elaborate on this item by providing additional information not in the ms, or briefly explain why the item is not applicable/relevant for your study

Yes, "Patients received an online questionnaire and an accelerometer at baseline and after 3 months of follow-up. Baseline measurement was conducted face to face and follow-up measurement through online communication, e.g., FaceTime, or face to face when requested."

4b-ii) Report how institutional affiliations are displayed

Report how institutional affiliations are displayed to potential participants [on ehealth media], as affiliations with prestigious hospitals or universities may affect volunteer rates, use, and reactions with regards to an intervention.(Not a required item – describe only if this may bias results)

	1	2	3	4	5	
subitem not at all important	(●)	( )	( )	( )	( )	essential

Selectie wissen

Does your paper address subitem 4b-ii?

Copy and paste relevant sections from the manuscript (include quotes in quotation marks "like this" to indicate direct quotes from your manuscript), or elaborate on this item by providing additional information not in the ms, or briefly explain why the item is not applicable/relevant for your study

Jouw antwoord

5) The interventions for each group with sufficient details to allow replication, including how and when they were actually administered

5-i) Mention names, credential, affiliations of the developers, sponsors, and owners

Mention names, credential, affiliations of the developers, sponsors, and owners [6] (if authors/evaluators are owners or developer of the software, this needs to be declared in a “Conflict of interest” section or mentioned elsewhere in the manuscript).

	1	2	3	4	5	
subitem not at all important	○	◉	○	○	○	essential

Selectie wissen

Does your paper address subitem 5-i?

Copy and paste relevant sections from the manuscript (include quotes in quotation marks "like this" to indicate direct quotes from your manuscript), or elaborate on this item by providing additional information not in the ms, or briefly explain why the item is not applicable/relevant for your study

No, see protocol study for more details.

5-ii) Describe the history/development process

Describe the history/development process of the application and previous formative evaluations (e.g., focus groups, usability testing), as these will have an impact on adoption/use rates and help with interpreting results.

	1	2	3	4	5	
subitem not at all important	○	○	◉	○	○	essential

Selectie wissen

Does your paper address subitem 5-ii?

Copy and paste relevant sections from the manuscript (include quotes in quotation marks "like this" to indicate direct quotes from your manuscript), or elaborate on this item by providing additional information not in the ms, or briefly explain why the item is not applicable/relevant for your study

Yes, "Therefore, we recently developed e-Exercise LBP, a stratified blended intervention in which a smartphone application is integrated within face-to-face physiotherapy treatment, and established its feasibility and proof of concept for treatment of functional disability and pain[23]. E-Exercise LBP is an adapted version of previously developed and evaluated blended physiotherapy programs[24,25]." Further details are given in the protocol study.

5-iii) Revisions and updating

Revisions and updating. Clearly mention the date and/or version number of the application/intervention (and comparator, if applicable) evaluated, or describe whether the intervention underwent major changes during the evaluation process, or whether the development and/or content was “frozen” during the trial. Describe dynamic components such as news feeds or changing content which may have an impact on the replicability of the intervention (for unexpected events see item 3b).

	1	2	3	4	5	
subitem not at all important	(•)	( )	( )	( )	( )	essential

Selectie wissen

Does your paper address subitem 5-iii?

Copy and paste relevant sections from the manuscript (include quotes in quotation marks "like this" to indicate direct quotes from your manuscript), or elaborate on this item by providing additional information not in the ms, or briefly explain why the item is not applicable/relevant for your study

Jouw antwoord

5-iv) Quality assurance methods

Provide information on quality assurance methods to ensure accuracy and quality of information provided [1], if applicable.

	1	2	3	4	5	
subitem not at all important	( )	( )	(•)	( )	( )	essential

Selectie wissen

Does your paper address subitem 5-iv?

Copy and paste relevant sections from the manuscript (include quotes in quotation marks "like this" to indicate direct quotes from your manuscript), or elaborate on this item by providing additional information not in the ms, or briefly explain why the item is not applicable/relevant for your study

Jouw antwoord

5-v) Ensure replicability by publishing the source code, and/or providing screenshots/screen-capture video, and/or providing flowcharts of the algorithms used

Ensure replicability by publishing the source code, and/or providing screenshots/screen-capture video, and/or providing flowcharts of the algorithms used. Replicability (i.e., other researchers should in principle be able to replicate the study) is a hallmark of scientific reporting.

	1	2	3	4	5	
subitem not at all important	○	◉	○	○	○	essential

Selectie wissen

Does your paper address subitem 5-v?

Copy and paste relevant sections from the manuscript (include quotes in quotation marks "like this" to indicate direct quotes from your manuscript), or elaborate on this item by providing additional information not in the ms, or briefly explain why the item is not applicable/relevant for your study

Yes, "Print screens of the smartphone application are given in Multimedia Appendix 2"

5-vi) Digital preservation

Digital preservation: Provide the URL of the application, but as the intervention is likely to change or disappear over the course of the years; also make sure the intervention is archived (Internet Archive, webcitation.org, and/or publishing the source code or screenshots/videos alongside the article). As pages behind login screens cannot be archived, consider creating demo pages which are accessible without login.

	1	2	3	4	5	
subitem not at all important	◉	○	○	○	○	essential

Selectie wissen

Does your paper address subitem 5-vi?

Copy and paste relevant sections from the manuscript (include quotes in quotation marks "like this" to indicate direct quotes from your manuscript), or elaborate on this item by providing additional information not in the ms, or briefly explain why the item is not applicable/relevant for your study

Jouw antwoord

5-vii) Access

Access: Describe how participants accessed the application, in what setting/context, if they had to pay (or were paid) or not, whether they had to be a member of specific group. If known, describe how participants obtained “access to the platform and Internet” [1]. To ensure access for editors/reviewers/readers, consider to provide a “backdoor” login account or demo mode for reviewers/readers to explore the application (also important for archiving purposes, see vi).

	1	2	3	4	5	
subitem not at all important	○	○	◉	○	○	essential

Selectie wissen

Does your paper address subitem 5-vii? *

Copy and paste relevant sections from the manuscript (include quotes in quotation marks "like this" to indicate direct quotes from your manuscript), or elaborate on this item by providing additional information not in the ms, or briefly explain why the item is not applicable/relevant for your study

Yes, "Both the content of face-to-face care and the smartphone application were tailored by the physiotherapists to the patients’ individual needs and progress (Table 1)."

5-viii) Mode of delivery, features/functionalities/components of the intervention and comparator, and the theoretical framework

Describe mode of delivery, features/functionalities/components of the intervention and comparator, and the theoretical framework [6] used to design them (instructional strategy [1], behaviour change techniques, persuasive features, etc., see e.g., [7, 8] for terminology). This includes an in-depth description of the content (including where it is coming from and who developed it) [1],” whether [and how] it is tailored to individual circumstances and allows users to track their progress and receive feedback” [6]. This also includes a description of communication delivery channels and – if computer-mediated communication is a component – whether communication was synchronous or asynchronous [6]. It also includes information on presentation strategies [1], including page design principles, average amount of text on pages, presence of hyperlinks to other resources, etc. [1].

	1	2	3	4	5	
subitem not at all important	○	○	○	◉	○	essential

Selectie wissen

Does your paper address subitem 5-viii? *

Copy and paste relevant sections from the manuscript (include quotes in quotation marks "like this" to indicate direct quotes from your manuscript), or elaborate on this item by providing additional information not in the ms, or briefly explain why the item is not applicable/relevant for your study

Yes, "Patients allocated to the stratified blended physiotherapy group received blended physiotherapy, consisting of a smartphone application integrated within face-to-face physiotherapy treatment[23,26]. Both the content of the smartphone application and the face-to-face physiotherapy treatment are based on the recommendations of the LBP guidelines of The Royal Dutch Society for Physiotherapy[11]. The duration and content of the stratified blended physiotherapy intervention was based on the patients' risk for developing persistent LBP ('low', 'medium' or 'high') using the Keele STarT Back Screening Tool[9,10]. The smartphone application contains video-supported self-management information, video-supported exercises and a goal-oriented physical activity module. Both the content of face-to-face care and the smartphone application were tailored by the physiotherapists to the patients' individual needs and progress (Table 1)."

5-ix) Describe use parameters

Describe use parameters (e.g., intended "doses" and optimal timing for use). Clarify what instructions or recommendations were given to the user, e.g., regarding timing, frequency, heaviness of use, if any, or was the intervention used ad libitum.

	1	2	3	4	5	
subitem not at all important	○	◉	○	○	○	essential

Selectie wissen

Does your paper address subitem 5-ix?

Copy and paste relevant sections from the manuscript (include quotes in quotation marks "like this" to indicate direct quotes from your manuscript), or elaborate on this item by providing additional information not in the ms, or briefly explain why the item is not applicable/relevant for your study

Jouw antwoord

5-x) Clarify the level of human involvement

Clarify the level of human involvement (care providers or health professionals, also technical assistance) in the e-intervention or as co-intervention (detail number and expertise of professionals involved, if any, as well as “type of assistance offered, the timing and frequency of the support, how it is initiated, and the medium by which the assistance is delivered”. It may be necessary to distinguish between the level of human involvement required for the trial, and the level of human involvement required for a routine application outside of a RCT setting (discuss under item 21 – generalizability).

	1	2	3	4	5	
subitem not at all important	○	○	◉	○	○	essential

Selectie wissen

Does your paper address subitem 5-x?

Copy and paste relevant sections from the manuscript (include quotes in quotation marks "like this" to indicate direct quotes from your manuscript), or elaborate on this item by providing additional information not in the ms, or briefly explain why the item is not applicable/relevant for your study

Yes, "Both the content of face-to-face care and the smartphone application were tailored by the physiotherapists to the patients’ individual needs and progress (Table 1)."

5-xi) Report any prompts/reminders used

Report any prompts/reminders used: Clarify if there were prompts (letters, emails, phone calls, SMS) to use the application, what triggered them, frequency etc. It may be necessary to distinguish between the level of prompts/reminders required for the trial, and the level of prompts/reminders for a routine application outside of a RCT setting (discuss under item 21 – generalizability).

	1	2	3	4	5	
subitem not at all important	○	○	◉	○	○	essential

Selectie wissen

Does your paper address subitem 5-xi? *

Copy and paste relevant sections from the manuscript (include quotes in quotation marks "like this" to indicate direct quotes from your manuscript), or elaborate on this item by providing additional information not in the ms, or briefly explain why the item is not applicable/relevant for your study

Yes, Extensive description is given in the protocol study and is referred to.

5-xii) Describe any co-interventions (incl. training/support)

Describe any co-interventions (incl. training/support): Clearly state any interventions that are provided in addition to the targeted eHealth intervention, as ehealth intervention may not be designed as stand-alone intervention. This includes training sessions and support [1]. It may be necessary to distinguish between the level of training required for the trial, and the level of training for a routine application outside of a RCT setting (discuss under item 21 – generalizability.

	1	2	3	4	5	
subitem not at all important	○	○	◉	○	○	essential

Selectie wissen

Does your paper address subitem 5-xii? *

Copy and paste relevant sections from the manuscript (include quotes in quotation marks "like this" to indicate direct quotes from your manuscript), or elaborate on this item by providing additional information not in the ms, or briefly explain why the item is not applicable/relevant for your study

Yes, "Patients allocated to the stratified blended physiotherapy group received blended physiotherapy, consisting of a smartphone application integrated within face-to-face physiotherapy treatment[23,26]. Both the content of the smartphone application and the face-to-face physiotherapy treatment are based on the recommendations of the LBP guidelines of The Royal Dutch Society for Physiotherapy[11]. The duration and content of the stratified blended physiotherapy intervention was based on the patients' risk for developing persistent LBP ('low', 'medium' or 'high') using the Keele STarT Back Screening Tool[9,10]. The smartphone application contains video-supported self-management information, video-supported exercises and a goal-oriented physical activity module. Both the content of face-to-face care and the smartphone application were tailored by the physiotherapists to the patients' individual needs and progress (Table 1)."

6a) Completely defined pre-specified primary and secondary outcome measures, including how and when they were assessed

Does your paper address CONSORT subitem 6a? *

Copy and paste relevant sections from the manuscript (include quotes in quotation marks "like this" to indicate direct quotes from your manuscript), or elaborate on this item by providing additional information not in the ms, or briefly explain why the item is not applicable/relevant for your study

Yes, "Outcome measures
Primary outcome
Physical functioning due to pain was assessed by the Oswestry Disability Index (ODI), version 2.1a[27,28]. The ODI was derived from the internationally accepted “Core Outcome Set” for research into patients with nonspecific LBP[28]. A higher score (0-100) indicates increased functional disability.

Secondary outcomes
Pain intensity was measured with an 11-point Numeric Rating Scale (NRS) for the average LBP intensity in the last week (0=no pain; 10=worst possible pain)[28,29].

Physical activity was objectively measured using the Activ8 (ACTIV8, Valkenswaard, The Netherlands)[30]. Patients were instructed to wear the Activ8 for five consecutive weeks starting at baseline and eight consecutive days at the 3-month follow-up, except during sleeping, showering, bathing or swimming. For the purpose of the current study, only the first seven days at both baseline and 3-month follow-up were used. Accelerometer data were eligible if patients had worn the metre for at least three days for ≥10 hours a day[31]. Per patient, the mean time spent in moderate to vigorous physical activity (MVPA) (all activities >3.0 Metabolic Equivalents[32]) in minutes per day were computed by summation and divided by the number of eligible wearing days.

Fear avoidance beliefs about physical activity and work were measured using the Fear-Avoidance Beliefs Questionnaire (FABQ)[33]. A higher score (range 0-96) indicates stronger fear and avoidance beliefs about how physical activity and work negatively affect LBP.

Pain catastrophizing was measured by the Pain Catastrophizing Scale (PCS)[34]. A higher score (range 0-55) indicates a higher level of catastrophizing.

Self-efficacy was measured using the General Self-efficacy Scale (GSE Scale)[35,36]. A higher score (range 10-40) indicates greater or stronger perceived self-efficacy.

Self-management ability was assessed through the Dutch version of the short form Patient Activation Measure (PAM 13-Dutch)[37]. A higher score (range 0-100) indicates a higher level of self-management.

Health-related quality of life was measured using the EuroQol-5D-5L (EQ-5D-5L)[38]. A higher score (range 0-100) indicates a higher health-related quality of life.
Patient self-reported adherence to prescribed home exercises was measured by the Exercise Adherence Rating Scale (EARS)[39]. A higher score (range 0-24) indicates better adherence."

And
"Patients received an online questionnaire and an accelerometer at baseline and after 3 months of follow-up. Baseline measurement was conducted face to face and follow-up measurement through online communication, e.g., FaceTime, or face to face when requested. No financial incentives were offered to complete the measurements. In the case of an unfilled questionnaire, patients were reminded after seven and fourteen days."

6a-i) Online questionnaires: describe if they were validated for online use and apply CHERRIES items to describe how the questionnaires were designed/deployed

If outcomes were obtained through online questionnaires, describe if they were validated for online use and apply CHERRIES items to describe how the questionnaires were designed/deployed [9].

	1	2	3	4	5	
subitem not at all important	◉	○	○	○	○	essential

Selectie wissen

Does your paper address subitem 6a-i?

Copy and paste relevant sections from manuscript text

Jouw antwoord

6a-ii) Describe whether and how “use” (including intensity of use/dosage) was defined/measured/monitored

Describe whether and how “use” (including intensity of use/dosage) was defined/measured/monitored (logins, logfile analysis, etc.). Use/adoption metrics are important process outcomes that should be reported in any ehealth trial.

	1	2	3	4	5	
subitem not at all important	○	○	◉	○	○	essential

Selectie wissen

Does your paper address subitem 6a-ii?

Copy and paste relevant sections from manuscript text

Jouw antwoord

6a-iii) Describe whether, how, and when qualitative feedback from participants was obtained

Describe whether, how, and when qualitative feedback from participants was obtained (e.g., through emails, feedback forms, interviews, focus groups).

	1	2	3	4	5	
subitem not at all important	○	○	◉	○	○	essential

Selectie wissen

Does your paper address subitem 6a-iii?

Copy and paste relevant sections from manuscript text

Jouw antwoord

6b) Any changes to trial outcomes after the trial commenced, with reasons

Does your paper address CONSORT subitem 6b? *

Copy and paste relevant sections from the manuscript (include quotes in quotation marks "like this" to indicate direct quotes from your manuscript), or elaborate on this item by providing additional information not in the ms, or briefly explain why the item is not applicable/relevant for your study

Not applicable, no changes were made.

7a) How sample size was determined

NPT: When applicable, details of whether and how the clustering by care provides or centers was addressed

7a-i) Describe whether and how expected attrition was taken into account when calculating the sample size

Describe whether and how expected attrition was taken into account when calculating the sample size.

	1	2	3	4	5	
subitem not at all important	○	◉	○	○	○	essential

Selectie wissen

Does your paper address subitem 7a-i?

Copy and paste relevant sections from manuscript title (include quotes in quotation marks "like this" to indicate direct quotes from your manuscript), or elaborate on this item by providing additional information not in the ms, or briefly explain why the item is not applicable/relevant for your study

Yes, but it does not focus on this, since study focuses on the effects of stratified blended care.

7b) When applicable, explanation of any interim analyses and stopping guidelines

Does your paper address CONSORT subitem 7b? *

Copy and paste relevant sections from the manuscript (include quotes in quotation marks "like this" to indicate direct quotes from your manuscript), or elaborate on this item by providing additional information not in the ms, or briefly explain why the item is not applicable/relevant for your study

Not Applicable, not conducted.

8a) Method used to generate the random allocation sequence

NPT: When applicable, how care providers were allocated to each trial group

Does your paper address CONSORT subitem 8a? *

Copy and paste relevant sections from the manuscript (include quotes in quotation marks "like this" to indicate direct quotes from your manuscript), or elaborate on this item by providing additional information not in the ms, or briefly explain why the item is not applicable/relevant for your study

Yes, "Physiotherapists were cluster-randomized on the level of the practice to avoid contamination. Treatment allocation was concealed and done by an independent researcher using a computer-generated, a priori created, random sequence table and a 1:1 allocation ratio. Physiotherapists and patients were not blind to group allocation."

8b) Type of randomisation; details of any restriction (such as blocking and block size)

Does your paper address CONSORT subitem 8b? *

Copy and paste relevant sections from the manuscript (include quotes in quotation marks "like this" to indicate direct quotes from your manuscript), or elaborate on this item by providing additional information not in the ms, or briefly explain why the item is not applicable/relevant for your study

Yes, "Physiotherapists were cluster-randomized on the level of the practice to avoid contamination. Treatment allocation was concealed and done by an independent researcher using a computer-generated, a priori created, random sequence table and a 1:1 allocation ratio. Physiotherapists and patients were not blind to group allocation."

9) Mechanism used to implement the random allocation sequence (such as sequentially numbered containers), describing any steps taken to conceal the sequence until interventions were assigned

Does your paper address CONSORT subitem 9? *

Copy and paste relevant sections from the manuscript (include quotes in quotation marks "like this" to indicate direct quotes from your manuscript), or elaborate on this item by providing additional information not in the ms, or briefly explain why the item is not applicable/relevant for your study

Yes, "Physiotherapists were cluster-randomized on the level of the practice to avoid contamination. Treatment allocation was concealed and done by an independent researcher using a computer-generated, a priori created, random sequence table and a 1:1 allocation ratio. Physiotherapists and patients were not blind to group allocation."

10) Who generated the random allocation sequence, who enrolled participants, and who assigned participants to interventions

Does your paper address CONSORT subitem 10? *

Copy and paste relevant sections from the manuscript (include quotes in quotation marks "like this" to indicate direct quotes from your manuscript), or elaborate on this item by providing additional information not in the ms, or briefly explain why the item is not applicable/relevant for your study

Yes, "Physiotherapists were cluster-randomized on the level of the practice to avoid contamination. Treatment allocation was concealed and done by an independent researcher using a computer-generated, a priori created, random sequence table and a 1:1 allocation ratio. Physiotherapists and patients were not blind to group allocation."

11a) If done, who was blinded after assignment to interventions (for example, participants, care providers, those assessing outcomes) and how

NPT: Whether or not administering co-interventions were blinded to group assignment

11a-i) Specify who was blinded, and who wasn't

Specify who was blinded, and who wasn't. Usually, in web-based trials it is not possible to blind the participants [1, 3] (this should be clearly acknowledged), but it may be possible to blind outcome assessors, those doing data analysis or those administering co-interventions (if any).

	1	2	3	4	5	
subitem not at all important	○	○	◉	○	○	essential

Selectie wissen

Does your paper address subitem 11a-i? *

Copy and paste relevant sections from the manuscript (include quotes in quotation marks "like this" to indicate direct quotes from your manuscript), or elaborate on this item by providing additional information not in the ms, or briefly explain why the item is not applicable/relevant for your study

Yes, "Physiotherapists were cluster-randomized on the level of the practice to avoid contamination. Treatment allocation was concealed and done by an independent researcher using a computer-generated, a priori created, random sequence table and a 1:1 allocation ratio. Physiotherapists and patients were not blind to group allocation."

11a-ii) Discuss e.g., whether participants knew which intervention was the “intervention of interest” and which one was the “comparator”

Informed consent procedures (4a-ii) can create biases and certain expectations - discuss e.g., whether participants knew which intervention was the “intervention of interest” and which one was the “comparator”.

	1	2	3	4	5	
subitem not at all important	○	○	○	◉	○	essential

Selectie wissen

Does your paper address subitem 11a-ii?

Copy and paste relevant sections from the manuscript (include quotes in quotation marks "like this" to indicate direct quotes from your manuscript), or elaborate on this item by providing additional information not in the ms, or briefly explain why the item is not applicable/relevant for your study

Yes, included in the patient information letter and informed consent procedure.

11b) If relevant, description of the similarity of interventions

(this item is usually not relevant for ehealth trials as it refers to similarity of a placebo or sham intervention to a active medication/intervention)

Does your paper address CONSORT subitem 11b? *

Copy and paste relevant sections from the manuscript (include quotes in quotation marks "like this" to indicate direct quotes from your manuscript), or elaborate on this item by providing additional information not in the ms, or briefly explain why the item is not applicable/relevant for your study

Yes, "Patients in the face-to-face physiotherapy group received only face-to-face care following the recommendations of the LBP guidelines of The Royal Dutch Society for Physiotherapy[11]. The guideline distinguishes between three different patient profiles based on the clinical course of recovery (i.e. normal recovery, abnormal recovery without predominant psychosocial factors, and abnormal recovery with predominant psychosocial factors), but does not use a specific tool to stratify care a priori. The content of face-to-face physiotherapy was the same as the stratified blended care intervention, i.e., information, exercises, and recommendations regarding physical activity."

12a) Statistical methods used to compare groups for primary and secondary outcomes

NPT: When applicable, details of whether and how the clustering by care providers or centers was addressed

Does your paper address CONSORT subitem 12a? *

Copy and paste relevant sections from the manuscript (include quotes in quotation marks "like this" to indicate direct quotes from your manuscript), or elaborate on this item by providing additional information not in the ms, or briefly explain why the item is not applicable/relevant for your study

Yes, "Linear mixed models (LMMs) with random effects to control for correlation within patients and physiotherapy practices[41] were used to determine the short-term effectiveness of stratified blended physiotherapy compared to face-to-face physiotherapy on primary and secondary outcome measures. Regression coefficients with 95% confidence intervals (CIs) signifying differences between stratified blended physiotherapy and face-to-face physiotherapy were estimated. Analyses were adjusted for predefined confounders (e.g., age, gender, duration of pain[42–44]) that changed the between-group estimate by ≥10%. In addition, analyses were also adjusted for variables with a substantial difference at baseline that changed the regression coefficient for the between-group estimate by ≥10%. Potential interaction terms were explored. In the case of a statistically significant interaction term, a stratified LMMs analyses, controlling for the same variables as the primary analysis, were performed for the effect modifier."

12a-i) Imputation techniques to deal with attrition / missing values

Imputation techniques to deal with attrition / missing values: Not all participants will use the intervention/comparator as intended and attrition is typically high in ehealth trials. Specify how participants who did not use the application or dropped out from the trial were treated in the statistical analysis (a complete case analysis is strongly discouraged, and simple imputation techniques such as LOCF may also be problematic [4]).

	1	2	3	4	5	
subitem not at all important	○	○	●	○	○	essential

Selectie wissen

Does your paper address subitem 12a-i? *

Copy and paste relevant sections from the manuscript (include quotes in quotation marks "like this" to indicate direct quotes from your manuscript), or elaborate on this item by providing additional information not in the ms, or briefly explain why the item is not applicable/relevant for your study

Yes, "Descriptive statistics were used to explore baseline comparability and to describe patients' general characteristics, the number of face-to-face physiotherapy sessions, and the treatment modalities. To investigate selective attrition, general characteristics and primary baseline variables of dropouts and nondropouts were compared. All analyses were performed according to the 'intention-to-treat' principle. Missing value analyses were performed by assuming the missing at random assumption. Multiple imputation was applied using 'Multivariate Imputation by Chained Equations' with Predictive Mean Matching for missing data in all outcomes. Thirty-six imputed datasets were generated corresponding to the highest missing value percentage[40]."

12b) Methods for additional analyses, such as subgroup analyses and adjusted analyses

Does your paper address CONSORT subitem 12b? *

Copy and paste relevant sections from the manuscript (include quotes in quotation marks "like this" to indicate direct quotes from your manuscript), or elaborate on this item by providing additional information not in the ms, or briefly explain why the item is not applicable/relevant for your study

Yes, "Analyses were adjusted for predefined confounders (e.g., age, gender, duration of pain[42–44]) that changed the between-group estimate by ≥10%. In addition, analyses were also adjusted for variables with a substantial difference at baseline that changed the regression coefficient for the between-group estimate by ≥10%. Potential interaction terms were explored. In the case of a statistically significant interaction term, a stratified LMMs analyses, controlling for the same variables as the primary analysis, were performed for the effect modifier."

X26) REB/IRB Approval and Ethical Considerations [recommended as subheading under "Methods"] (not a CONSORT item)

X26-i) Comment on ethics committee approval

	1	2	3	4	5	
subitem not at all important	○	○	◉	○	○	essential

Selectie wissen

Does your paper address subitem X26-i?

Copy and paste relevant sections from the manuscript (include quotes in quotation marks "like this" to indicate direct quotes from your manuscript), or elaborate on this item by providing additional information not in the ms, or briefly explain why the item is not applicable/relevant for your study

Yes, "The study protocol was approved by the Medical Research Ethics Committee of the University Medical Center Utrecht, the Netherlands (18-085/D), and registered at the onset of patient enrolment (ISRCTN 94074203). "

x26-ii) Outline informed consent procedures

Outline informed consent procedures e.g., if consent was obtained offline or online (how? Checkbox, etc.?), and what information was provided (see 4a-ii). See [6] for some items to be included in informed consent documents.

	1	2	3	4	5	
subitem not at all important	○	○	○	◉	○	essential

Selectie wissen

Does your paper address subitem X26-ii?

Copy and paste relevant sections from the manuscript (include quotes in quotation marks "like this" to indicate direct quotes from your manuscript), or elaborate on this item by providing additional information not in the ms, or briefly explain why the item is not applicable/relevant for your study

Yes, "When a patient was willing to participate after the phone call, a face-to-face appointment was scheduled (by TK or RA) to obtain written informed consent and verify eligibility."

X26-iii) Safety and security procedures

Safety and security procedures, incl. privacy considerations, and any steps taken to reduce the likelihood or detection of harm (e.g., education and training, availability of a hotline)

	1	2	3	4	5	
subitem not at all important	○	○	◉	○	○	essential

Selectie wissen

Does your paper address subitem X26-iii?

Copy and paste relevant sections from the manuscript (include quotes in quotation marks "like this" to indicate direct quotes from your manuscript), or elaborate on this item by providing additional information not in the ms, or briefly explain why the item is not applicable/relevant for your study

Yes, "Physiotherapists in the stratified blended physiotherapy group received two 4-hour training sessions about e-Exercise LBP and the study procedures. In the face-to-face physiotherapy group, physiotherapists received one 4-hour training session in current best practices according to the LBP guidelines of The Royal Dutch Society for Physiotherapy[11] and the study procedures."

RESULTS

13a) For each group, the numbers of participants who were randomly assigned, received intended treatment, and were analysed for the primary outcome

NPT: The number of care providers or centers performing the intervention in each group and the number of patients treated by each care provider in each center

Does your paper address CONSORT subitem 13a? *

Copy and paste relevant sections from the manuscript (include quotes in quotation marks "like this" to indicate direct quotes from your manuscript), or elaborate on this item by providing additional information not in the ms, or briefly explain why the item is not applicable/relevant for your study

Yes, "From June 2018 to December 2019, 434 possible eligible patients with LBP were asked to participate in 58 physiotherapy practices. In 22 physiotherapy practices allocated to stratified blended physiotherapy and 20 practices allocated to face-to-face physiotherapy, 208 (47.9%) patients were included (Figure 1).
Baseline characteristics of patients are presented in Table 2. The stratified blended physiotherapy group consisted of more males, more patients with a low level of education, and more patients with a duration of LBP >12 months. No other relevant differences in characteristics were seen between groups. At baseline, complete data on outcome measures were available from 97.1% of the patients in the stratified blended physiotherapy group and 99.0% of the patients in the face-to-face physiotherapy group and eligible accelerometer data were available from 84.6% and 83.7%, respectively Four ineligible patients (n=2 stratified blended physiotherapy; n=2 face-to-face physiotherapy) who were unjustified included, did not receive the allocated intervention and were therefore excluded from all analyses.
At the 3-month follow-up, complete data on outcome measures were available from 86.5% of the patients in the stratified blended physiotherapy group and 93.3% of the patients in the face-to-face physiotherapy group, and eligible accelerometer data were available from 74.0% and 76.0% of these patients, respectively."

13b) For each group, losses and exclusions after randomisation, together with reasons

Does your paper address CONSORT subitem 13b? (NOTE: Preferably, this is shown in a CONSORT flow diagram) *

Copy and paste relevant sections from the manuscript (include quotes in quotation marks "like this" to indicate direct quotes from your manuscript), or elaborate on this item by providing additional information not in the ms, or briefly explain why the item is not applicable/relevant for your study

Yes, Shown in the flow diagram and "At baseline, complete data on outcome measures were available from 97.1% of the patients in the stratified blended physiotherapy group and 99.0% of the patients in the face-to-face physiotherapy group and eligible accelerometer data were available from 84.6% and 83.7%, respectively Four ineligible patients (n=2 stratified blended physiotherapy; n=2 face-to-face physiotherapy) who were unjustified included, did not receive the allocated intervention and were therefore excluded from all analyses.
At the 3-month follow-up, complete data on outcome measures were available from 86.5% of the patients in the stratified blended physiotherapy group and 93.3% of the patients in the face-to-face physiotherapy group, and eligible accelerometer data were available from 74.0% and 76.0% of these patients, respectively."

13b-i) Attrition diagram

Strongly recommended: An attrition diagram (e.g., proportion of participants still logging in or using the intervention/comparator in each group plotted over time, similar to a survival curve) or other figures or tables demonstrating usage/dose/engagement.

	1	2	3	4	5	
subitem not at all important	○	◉	○	○	○	essential

Selectie wissen

Does your paper address subitem 13b-i?

Copy and paste relevant sections from the manuscript or cite the figure number if applicable (include quotes in quotation marks "like this" to indicate direct quotes from your manuscript), or elaborate on this item by providing additional information not in the ms, or briefly explain why the item is not applicable/relevant for your study

Jouw antwoord

14a) Dates defining the periods of recruitment and follow-up

Does your paper address CONSORT subitem 14a? *

Copy and paste relevant sections from the manuscript (include quotes in quotation marks "like this" to indicate direct quotes from your manuscript), or elaborate on this item by providing additional information not in the ms, or briefly explain why the item is not applicable/relevant for your study

Yes, "From June 2018 to December 2019"

14a-i) Indicate if critical “secular events” fell into the study period

Indicate if critical “secular events” fell into the study period, e.g., significant changes in Internet resources available or “changes in computer hardware or Internet delivery resources”

	1	2	3	4	5	
subitem not at all important	○	○	○	○	○	essential

Does your paper address subitem 14a-i?

Copy and paste relevant sections from the manuscript (include quotes in quotation marks "like this" to indicate direct quotes from your manuscript), or elaborate on this item by providing additional information not in the ms, or briefly explain why the item is not applicable/relevant for your study

Jouw antwoord

14b) Why the trial ended or was stopped (early)

Does your paper address CONSORT subitem 14b? *

Copy and paste relevant sections from the manuscript (include quotes in quotation marks "like this" to indicate direct quotes from your manuscript), or elaborate on this item by providing additional information not in the ms, or briefly explain why the item is not applicable/relevant for your study

No, Not applicable for this study, inclusion was sufficient.

15) A table showing baseline demographic and clinical characteristics for each group

NPT: When applicable, a description of care providers (case volume, qualification, expertise, etc.) and centers (volume) in each group

Does your paper address CONSORT subitem 15? *

Copy and paste relevant sections from the manuscript (include quotes in quotation marks "like this" to indicate direct quotes from your manuscript), or elaborate on this item by providing additional information not in the ms, or briefly explain why the item is not applicable/relevant for your study

Yes, see table 2 and in text: "Baseline characteristics of patients are presented in Table 2. The stratified blended physiotherapy group consisted of more males, more patients with a low level of education, and more patients with a duration of LBP >12 months. No other relevant differences in characteristics were seen between groups."

15-i) Report demographics associated with digital divide issues

In ehealth trials it is particularly important to report demographics associated with digital divide issues, such as age, education, gender, social-economic status, computer/Internet/ehealth literacy of the participants, if known.

	1	2	3	4	5	
subitem not at all important	○	○	●	○	○	essential

Selectie wissen

Does your paper address subitem 15-i? *

Copy and paste relevant sections from the manuscript (include quotes in quotation marks "like this" to indicate direct quotes from your manuscript), or elaborate on this item by providing additional information not in the ms, or briefly explain why the item is not applicable/relevant for your study

No, since the focus is on startified blended care, which is an integration of an app within face-to-face care.

16) For each group, number of participants (denominator) included in each analysis and whether the analysis was by original assigned groups

16-i) Report multiple “denominators” and provide definitions

Report multiple “denominators” and provide definitions: Report N’s (and effect sizes) “across a range of study participation [and use] thresholds” [1], e.g., N exposed, N consented, N used more than x times, N used more than y weeks, N participants “used” the intervention/comparator at specific pre-defined time points of interest (in absolute and relative numbers per group). Always clearly define “use” of the intervention.

	1	2	3	4	5	
subitem not at all important	○	○	●	○	○	essential

Selectie wissen

Does your paper address subitem 16-i? *

Copy and paste relevant sections from the manuscript (include quotes in quotation marks "like this" to indicate direct quotes from your manuscript), or elaborate on this item by providing additional information not in the ms, or briefly explain why the item is not applicable/relevant for your study

Yes, see table 2-5 for the samples during analysis.

16-ii) Primary analysis should be intent-to-treat

Primary analysis should be intent-to-treat, secondary analyses could include comparing only “users”, with the appropriate caveats that this is no longer a randomized sample (see 18-i).

	1	2	3	4	5	
subitem not at all important	○	○	◉	○	○	essential

Selectie wissen

Does your paper address subitem 16-ii?

Copy and paste relevant sections from the manuscript (include quotes in quotation marks "like this" to indicate direct quotes from your manuscript), or elaborate on this item by providing additional information not in the ms, or briefly explain why the item is not applicable/relevant for your study

Jouw antwoord

17a) For each primary and secondary outcome, results for each group, and the estimated effect size and its precision (such as 95% confidence interval)

Does your paper address CONSORT subitem 17a? *

Copy and paste relevant sections from the manuscript (include quotes in quotation marks "like this" to indicate direct quotes from your manuscript), or elaborate on this item by providing additional information not in the ms, or briefly explain why the item is not applicable/relevant for your study

Yes, "In the mixed model analyses, log likelihood ratios of naïve models and models including a random intercept for both physiotherapy practice and physiotherapist were similar. Physiotherapy practice or physiotherapist was therefore not included as a level in the LMMs analyses. At 3 months, LMMs analyses showed no clinically relevant or statistically significant between-group difference in the primary outcome of physical functioning (mean difference (MD) -1.96; 95% CI, -4.47 to 0.55). For the secondary outcomes, a statistically significant between-group difference was found in favour of stratified blended physiotherapy for fear avoidance beliefs (MD -4.29; 95% CI, -7.22 to -1.37) and patient self-reported adherence to prescribed home exercises (MD 0.73; 95% CI, 0.06 to 1.39). Within-group analyses showed clinically relevant and statistically significant improvements in physical functioning (MD -11.48; 95% CI, -15.06 to -7.91), average pain intensity (MD -2.38; 95% CI, -3.00 to -1.76), and fear avoidance beliefs (MD -5.14; 95% CI, -9.22 to -1.06) in the stratified blended physiotherapy group. In the face-to-face physiotherapy group, clinically relevant and statistically significant improvements in physical functioning (MD -11.22; 95% CI, -14.64 to -7.80) and average pain intensity (MD -2.51; 95% CI, -3.11 to -1.90) were found (Table 4).

As indicated by a statistically significant interaction term, the patients' risk of developing persistent LBP was an effect modifier of the between-group differences on the primary outcome of physical functioning. In patients with a high risk of developing persistent LBP, the stratified analysis showed a statistically significant between-group difference in favour of stratified blended physiotherapy on physical functioning (MD -16.39; 95% CI, -27.98 to -4.79), average pain intensity (MD -3.43; 95% CI, -6.55 to -0.31), and fear avoidance beliefs (MD -14.51; 95% CI, -28.21 to -0.81). In patients with a medium risk of developing persistent LBP, a statistically significant between-group difference was found in favour of stratified blended physiotherapy on fear avoidance beliefs (MD -5.93; 95% CI, -11.45 to -0.40). In patients with a low risk of developing persistent LBP, no statistically significant between-group differences were found (Table 5)."

And table 4-5

17a-i) Presentation of process outcomes such as metrics of use and intensity of use

In addition to primary/secondary (clinical) outcomes, the presentation of process outcomes such as metrics of use and intensity of use (dose, exposure) and their operational definitions is critical. This does not only refer to metrics of attrition (13-b) (often a binary variable), but also to more continuous exposure metrics such as “average session length”. These must be accompanied by a technical description how a metric like a “session” is defined (e.g., timeout after idle time) [1] (report under item 6a).

	1	2	3	4	5	
subitem not at all important	○	◉	○	○	○	essential

Selectie wissen

Does your paper address subitem 17a-i?

Copy and paste relevant sections from the manuscript (include quotes in quotation marks "like this" to indicate direct quotes from your manuscript), or elaborate on this item by providing additional information not in the ms, or briefly explain why the item is not applicable/relevant for your study

Jouw antwoord

17b) For binary outcomes, presentation of both absolute and relative effect sizes is recommended

Does your paper address CONSORT subitem 17b? *

Copy and paste relevant sections from the manuscript (include quotes in quotation marks "like this" to indicate direct quotes from your manuscript), or elaborate on this item by providing additional information not in the ms, or briefly explain why the item is not applicable/relevant for your study

Not applicable, no binary outcomes

18) Results of any other analyses performed, including subgroup analyses and adjusted analyses, distinguishing pre-specified from exploratory

Does your paper address CONSORT subitem 18? *

Copy and paste relevant sections from the manuscript (include quotes in quotation marks "like this" to indicate direct quotes from your manuscript), or elaborate on this item by providing additional information not in the ms, or briefly explain why the item is not applicable/relevant for your study

Yes, "As indicated by a statistically significant interaction term, the patients' risk of developing persistent LBP was an effect modifier of the between-group differences on the primary outcome of physical functioning. In patients with a high risk of developing persistent LBP, the stratified analysis showed a statistically significant between-group difference in favour of stratified blended physiotherapy on physical functioning (MD -16.39; 95% CI, -27.98 to -4.79), average pain intensity (MD -3.43; 95% CI, -6.55 to -0.31), and fear avoidance beliefs (MD -14.51; 95% CI, -28.21 to -0.81). In patients with a medium risk of developing persistent LBP, a statistically significant between-group difference was found in favour of stratified blended physiotherapy on fear avoidance beliefs (MD -5.93; 95% CI, -11.45 to -0.40). In patients with a low risk of developing persistent LBP, no statistically significant between-group differences were found (Table 5)."

18-i) Subgroup analysis of comparing only users

A subgroup analysis of comparing only users is not uncommon in ehealth trials, but if done, it must be stressed that this is a self-selected sample and no longer an unbiased sample from a randomized trial (see 16-iii).

	1	2	3	4	5	
subitem not at all important	○	●	○	○	○	essential

Selectie wissen

Does your paper address subitem 18-i?

Copy and paste relevant sections from the manuscript (include quotes in quotation marks "like this" to indicate direct quotes from your manuscript), or elaborate on this item by providing additional information not in the ms, or briefly explain why the item is not applicable/relevant for your study

Jouw antwoord

19) All important harms or unintended effects in each group

(for specific guidance see CONSORT for harms)

Does your paper address CONSORT subitem 19? *

Copy and paste relevant sections from the manuscript (include quotes in quotation marks "like this" to indicate direct quotes from your manuscript), or elaborate on this item by providing additional information not in the ms, or briefly explain why the item is not applicable/relevant for your study

Not reported, since they did not occur.

19-i) Include privacy breaches, technical problems

Include privacy breaches, technical problems. This does not only include physical “harm” to participants, but also incidents such as perceived or real privacy breaches [1], technical problems, and other unexpected/unintended incidents. “Unintended effects” also includes unintended positive effects [2].

	1	2	3	4	5	
subitem not at all important	○	◉	○	○	○	essential

Selectie wissen

Does your paper address subitem 19-i?

Copy and paste relevant sections from the manuscript (include quotes in quotation marks "like this" to indicate direct quotes from your manuscript), or elaborate on this item by providing additional information not in the ms, or briefly explain why the item is not applicable/relevant for your study

Jouw antwoord

19-ii) Include qualitative feedback from participants or observations from staff/researchers

Include qualitative feedback from participants or observations from staff/researchers, if available, on strengths and shortcomings of the application, especially if they point to unintended/unexpected effects or uses. This includes (if available) reasons for why people did or did not use the application as intended by the developers.

	1	2	3	4	5	
subitem not at all important	○	◉	○	○	○	essential

Selectie wissen

Does your paper address subitem 19-ii?

Copy and paste relevant sections from the manuscript (include quotes in quotation marks "like this" to indicate direct quotes from your manuscript), or elaborate on this item by providing additional information not in the ms, or briefly explain why the item is not applicable/relevant for your study

Jouw antwoord

DISCUSSION

22) Interpretation consistent with results, balancing benefits and harms, and considering other relevant evidence

NPT: In addition, take into account the choice of the comparator, lack of or partial blinding, and unequal expertise of care providers or centers in each group

22-i) Restate study questions and summarize the answers suggested by the data, starting with primary outcomes and process outcomes (use)

Restate study questions and summarize the answers suggested by the data, starting with primary outcomes and process outcomes (use).

	1	2	3	4	5	
subitem not at all important	○	○	◉	○	○	essential

Selectie wissen

Does your paper address subitem 22-i? *

Copy and paste relevant sections from the manuscript (include quotes in quotation marks "like this" to indicate direct quotes from your manuscript), or elaborate on this item by providing additional information not in the ms, or briefly explain why the item is not applicable/relevant for your study

Yes, "This study evaluated the short-term (3 months) effectiveness of the stratified blended physiotherapy intervention e-Exercise LBP on physical functioning in comparison to face-to-face physiotherapy in patients with nonspecific LBP. In contrast to our expectations, the study results show no statistically significant between-group difference in physical functioning and most of the secondary outcome measures. Only fear avoidance beliefs and patient self-reported adherence to prescribed home exercises improved significantly in patients who were allocated to stratified blended physiotherapy. When looking at the different prognostic risk groups, in patients with a high risk of developing persistent LBP, a statistically significant between-group difference in favour of stratified blended physiotherapy on physical functioning, average pain intensity, and fear avoidance beliefs was found, but these results come with quite some uncertainty."

22-ii) Highlight unanswered new questions, suggest future research

Highlight unanswered new questions, suggest future research.

	1	2	3	4	5	
subitem not at all important	○	○	◉	○	○	essential

Selectie wissen

Does your paper address subitem 22-ii?

Copy and paste relevant sections from the manuscript (include quotes in quotation marks "like this" to indicate direct quotes from your manuscript), or elaborate on this item by providing additional information not in the ms, or briefly explain why the item is not applicable/relevant for your study

Yes, "Thus, looking at the different characteristics of online applications, such as the role of the healthcare professional within the intervention and the delivery mode and duration, future research needs to focus on the comparison of online applications with different characteristics, to obtain a better understanding of which elements work the best." and "Forthcoming studies need to determine which patients benefit most from a stratified blended physiotherapy approach." and "Further research on the long-term clinical relevance of adherence to home exercises as prescribed in e-Exercise LBP is ongoing." and "Future research should focus on determining whether this concerns the added value of the tool itself, or the added value of a stratified care approach in general." and " For future studies that aim to investigate postintervention effectiveness, it is therefore recommended to measure the clinical outcomes immediately after the intervention is finished and to monitor the time to recovery."

20) Trial limitations, addressing sources of potential bias, imprecision, and, if relevant, multiplicity of analyses

20-i) Typical limitations in ehealth trials

Typical limitations in ehealth trials: Participants in ehealth trials are rarely blinded. Ehealth trials often look at a multiplicity of outcomes, increasing risk for a Type I error. Discuss biases due to non-use of the intervention/usability issues, biases through informed consent procedures, unexpected events.

	1	2	3	4	5	
subitem not at all important	○	○	●	○	○	essential

Selectie wissen

Does your paper address subitem 20-i? *

Copy and paste relevant sections from the manuscript (include quotes in quotation marks "like this" to indicate direct quotes from your manuscript), or elaborate on this item by providing additional information not in the ms, or briefly explain why the item is not applicable/relevant for your study

Yes, "Nevertheless, this study also had a few limitations. First, the results seem to suggest that patients' risk of developing persistent LBP could be an effect modifier of the between-group differences on the primary outcome. Especially in the highest-risk group, consistent between-group differences were seen in both the primary and secondary outcomes, supporting the rationale for stratified blended physiotherapy. Since it was not the primary aim of this study, the sample size calculation did not take interaction into account, numbers were small, and results should therefore be interpreted with caution. Second, since we conducted a pragmatic study, the experiences of physiotherapists in either using online applications or treating patients with nonspecific LBP were not considered inclusion criteria for physiotherapy practices. However, given both the complexity of blended care[17] and the complexity of treating patients with nonspecific LBP[4], it can be expected that more experienced physiotherapists are able to deliver better treatment than less experienced physiotherapists. Therefore, experience might have influenced our analysis. Finally, four included patients were excluded from the analysis after being diagnosed with specific LBP. Since this number is low and occurred equally in both treatment groups (n=2), we expect that this has not influenced the results[64]."

21) Generalisability (external validity, applicability) of the trial findings

NPT: External validity of the trial findings according to the intervention, comparators, patients, and care providers or centers involved in the trial

21-i) Generalizability to other populations

Generalizability to other populations: In particular, discuss generalizability to a general Internet population, outside of a RCT setting, and general patient population, including applicability of the study results for other organizations

	1	2	3	4	5	
subitem not at all important	○	○	◉	○	○	essential

Selectie wissen

Does your paper address subitem 21-i?

Copy and paste relevant sections from the manuscript (include quotes in quotation marks "like this" to indicate direct quotes from your manuscript), or elaborate on this item by providing additional information not in the ms, or briefly explain why the item is not applicable/relevant for your study

Yes, " The baseline characteristics of both treatment groups and the distribution of the different prognostic risk groups of developing persistent LBP reflect the characteristics of patients with LBP normally being treated in primary care physiotherapy[60], which enhances the generalizability of our results."

21-ii) Discuss if there were elements in the RCT that would be different in a routine application setting

Discuss if there were elements in the RCT that would be different in a routine application setting (e.g., prompts/reminders, more human involvement, training sessions or other co-interventions) and what impact the omission of these elements could have on use, adoption, or outcomes if the intervention is applied outside of a RCT setting.

	1	2	3	4	5	
subitem not at all important	○	○	◉	○	○	essential

Selectie wissen

Does your paper address subitem 21-ii?

Copy and paste relevant sections from the manuscript (include quotes in quotation marks "like this" to indicate direct quotes from your manuscript), or elaborate on this item by providing additional information not in the ms, or briefly explain why the item is not applicable/relevant for your study

Yes, "The pragmatic multicentre cluster randomized controlled trial design allowed an evaluation of stratified blended physiotherapy in comparison to face-to-face physiotherapy in a real-world situation."

OTHER INFORMATION

23) Registration number and name of trial registry

Does your paper address CONSORT subitem 23? *

Copy and paste relevant sections from the manuscript (include quotes in quotation marks "like this" to indicate direct quotes from your manuscript), or elaborate on this item by providing additional information not in the ms, or briefly explain why the item is not applicable/relevant for your study

Yes this is mentioned in the method, "(ISRCTN 94074203)"

24) Where the full trial protocol can be accessed, if available

Does your paper address CONSORT subitem 24? *

Cite a Multimedia Appendix, other reference, or copy and paste relevant sections from the manuscript (include quotes in quotation marks "like this" to indicate direct quotes from your manuscript), or elaborate on this item by providing additional information not in the ms, or briefly explain why the item is not applicable/relevant for your study

Yes, "20. Koppenaal T, Arensman RM, Van Dongen JM, Ostelo RWJG, Veenhof C, Kloek CJJ, et al. Effectiveness and cost-effectiveness of stratified blended physiotherapy in patients with non-specific low back pain: Study protocol of a cluster randomized controlled trial. BMC Musculoskelet Disord 2020;21. https://doi.org/10.1186/s12891-020-3174-z."

25) Sources of funding and other support (such as supply of drugs), role of funders

Does your paper address CONSORT subitem 25? *

Copy and paste relevant sections from the manuscript (include quotes in quotation marks "like this" to indicate direct quotes from your manuscript), or elaborate on this item by providing additional information not in the ms, or briefly explain why the item is not applicable/relevant for your study

Yes, "This study was co-funded by the Taskforce for Applied Research SIA (RAAK.PRO02.063), part of the Dutch Research Council (NWO). The funder had no role in the study design, data collection and analysis, decision to publish, or preparation of the manuscript."

X27) Conflicts of Interest (not a CONSORT item)

X27-i) State the relation of the study team towards the system being evaluated

In addition to the usual declaration of interests (financial or otherwise), also state the relation of the study team towards the system being evaluated, i.e., state if the authors/evaluators are distinct from or identical with the developers/sponsors of the intervention.

	1	2	3	4	5	
subitem not at all important	○	○	●	○	○	essential

Selectie wissen

Does your paper address subitem X27-i?

Copy and paste relevant sections from the manuscript (include quotes in quotation marks "like this" to indicate direct quotes from your manuscript), or elaborate on this item by providing additional information not in the ms, or briefly explain why the item is not applicable/relevant for your study

Jouw antwoord

About the CONSORT EHEALTH checklist

As a result of using this checklist, did you make changes in your manuscript? *

- ○ yes, major changes
- ○ yes, minor changes
- ◉ no

What were the most important changes you made as a result of using this checklist?

Jouw antwoord

How much time did you spend on going through the checklist INCLUDING making changes in your manuscript *

I spent 1 hour to go through this checklist.

As a result of using this checklist, do you think your manuscript has improved? *

- ○ yes
- ◉ no
- ○ Anders:

Would you like to become involved in the CONSORT EHEALTH group?

This would involve for example becoming involved in participating in a workshop and writing an "Explanation and Elaboration" document

- ○ yes
- ◉ no
- ○ Anders:

Selectie wissen

Any other comments or questions on CONSORT EHEALTH

25 characters might be too long. Questions double on info.

STOP - Save this form as PDF before you click submit

To generate a record that you filled in this form, we recommend to generate a PDF of this page (on a Mac, simply select "print" and then select "print as PDF") before you submit it.

When you submit your (revised) paper to JMIR, please upload the PDF as supplementary file.

Don't worry if some text in the textboxes is cut off, as we still have the complete information in our database. Thank you!

Final step: Click submit !

Click submit so we have your answers in our database!

Verzenden Formulier wissen

Verzend nooit wachtwoorden via Google Formulieren.

Deze content is niet gemaakt of goedgekeurd door Google. Misbruik rapporteren - Servicevoorwaarden - Privacybeleid

Google Formulieren
