# Supplementary figures and images for: The 3-Month Effectiveness of a Stratified Blended Physiotherapy Intervention in Patients With Nonspecific Low Back Pain: Cluster Randomized Controlled Trial
Source: J Med Internet Res. 2022 Feb 25;24(2):e31675. doi: 10.2196/31675 (PMC8917429; doi:10.2196/31675)

## Multimedia Appendix 2. Print screens of the smartphone application

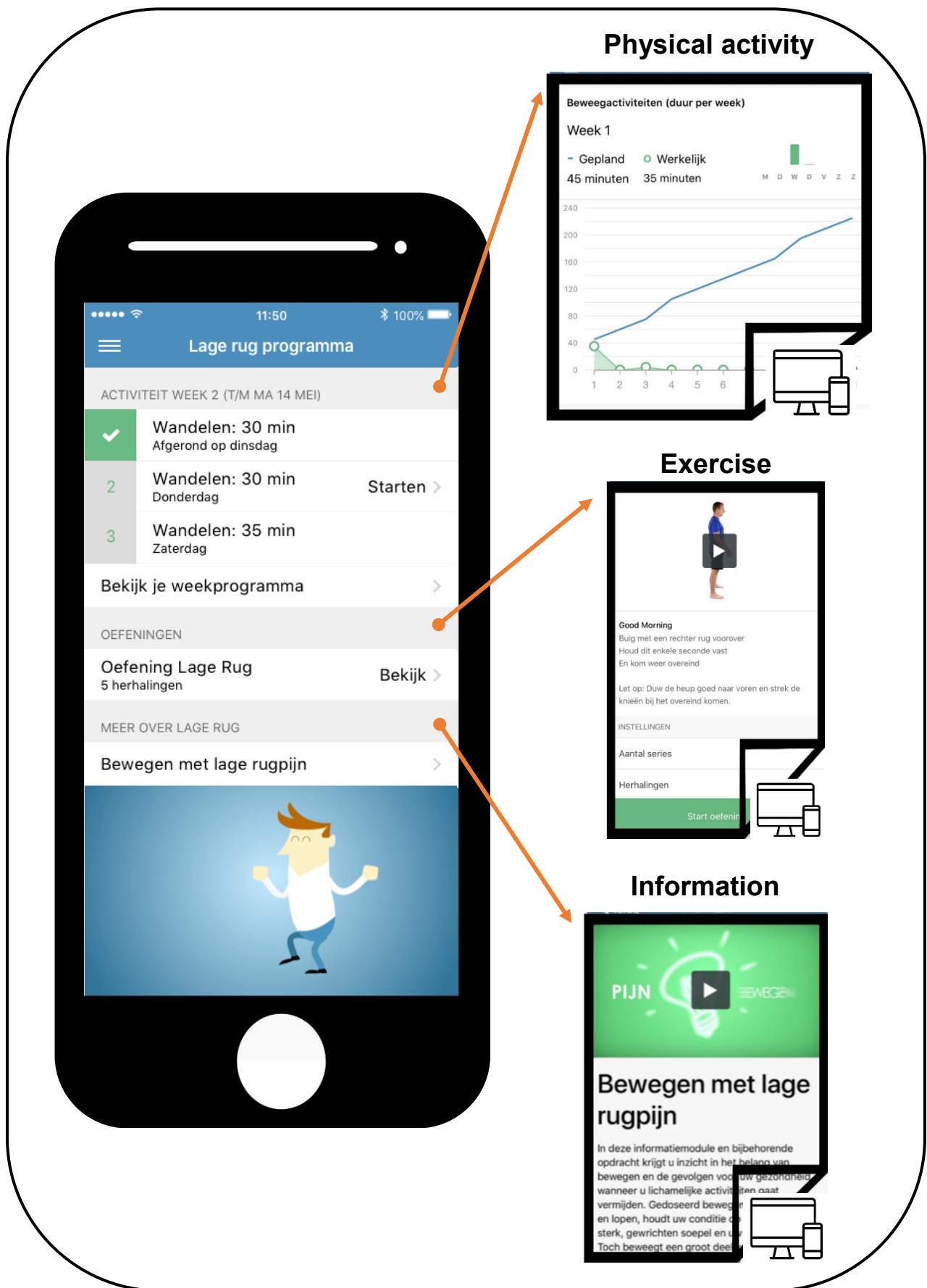

Supplement: Multimedia Appendix 2 [file jmir_v24i2e31675_app2.pdf]
